# Stimulus‐dependent deliberation process in left‐ and right‐handers obtained via current source density analysis

**DOI:** 10.14814/phy2.15522

**Published:** 2022-12-05

**Authors:** Florian P. Kolb, Dieter F. Kutz, Jana Werner, Sonja Schönecker, Walter Hürster, Julian Nida‐Rümelin

**Affiliations:** ^1^ Department of Physiology Physiological Genomics Faculty of Medicine Ludwig‐Maximilians‐University of Munich Munich Germany; ^2^ Department of Neuromotor Behavior and Exercise Institute of Sport and Exercise Sciences, University of Muenster Muenster Germany; ^3^ Department of Neurology University Hospital Zürich Zürich Switzerland; ^4^ Department of Neurology Ludwig‐Maximilians‐University of Munich Munich Germany; ^5^ Research and Consulting Ulm Germany; ^6^ Parmenides Foundation Pöcking Germany

**Keywords:** choice reaction task, color‐word‐Stroop task, comparison of right‐handed and left‐handed subjects, deliberation task, free will, high density EEG, Libet, movement intention, readiness potential

## Abstract

The aim of the present study was to compare the activity patterns of young, healthy right‐ (RH, *n* = 25) and left‐handed (LH, *n* = 20) subjects in high‐density electroencephalograpic (EEG) recordings during a deliberation task. The deliberation task consisted of pressing one of two keys depending on a color‐word Stroop task (Stroop, 1935) presented on a computer screen. Depending on the color shown and the meaning of the color word, participants responded with the index finger of the dominant or non‐dominant hand. This leads to different activities in the hemispheres depending on the acting hand and on subject's handedness. Presenting the word “black” in black color, subjects were not to press any key (no‐go‐trial). Prior to this, subjects were tested for simple motor tasks, during which they were informed about the motor action to be performed. The temporal activity patterns obtained from RH and LH were very similar in shape and constituent components. The comparison of the three types of trials lead to the assumption that the deliberation process is based on a two‐step decision: The first decision was characterized by the choice between move (match‐trials, mismatch‐trials) or not to move (no‐go‐trials). The second decision resulted in the final judgment of which index finger has to be used. The latter decision, in particular, can be tracked via the local spread of activity over the scalp. Our hypothesis is based on a comparison of activities and locations of RH and LH and yields some insights about processing a two‐step decision in a deliberation task.

## Graphical abstract

Stimulus‐dependent deliberation process based on a color‐word Stroop task (Stroop, 1935) in left‐ and right‐handers obtained via current source density analysis.

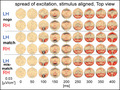

## 1 INTRODUCTION

### 1.1 Philosophical aspects

The results of the Libet experiment (Libet, 1985) and some follow‐up experiments (Fried et al., 2011; Soon et al., 2008) have had a major impact on how humans interpret themselves. These results have been taken widely as showing that the human self‐image is fundamentally wrong, that human action is not preceded by intentional decisions, but follows determinate brain processes that cannot be intentionally controlled by the agent, whereas Libet himself tried to uphold a minimal version of Free Will, that rests on the human capacity to veto ongoing brain processes that precede motor acts till 100 ms before the potential act would result (Libet, 1999, 2005). Some philosophers have criticized the interpretation of the Libet study (Schlosser, 2012; Zhu, 2003). Most philosophers assume that the agent's prior intentionality is constitutive for human agency (Mele, 1992; Nida‐Rümelin, 2022).

If the readiness potential (RP) starts before the decision is taken, the decision comes ex post, i.e., after the causal brain process leading to a motor act already has started. Intentions and deliberations that may speak in favor of or against an action then seem to be irrelevant for the action itself, they merely contribute to an ex‐post rationalization or identification of the acting person: qua rationalization the agents can present reasons if asked why they acted in such a way and they can identify themselves with their actions. Agency is an important part of the human self‐image—albeit erroneously, if the results of the Libet experiment and similar studies are not misleading. Our empirical findings speak for the thesis, that indeed these results and their interpretations are misleading.

The arrangement of the Libet study includes elements that are not easily combined: The participants are asked not to decide in advance when to move their hands. In other words, they are expected not to have the intention to move their hands at a certain time during the 30‐s interval. On the other they are asked to move their hands within the 30‐s interval. Therefore, there are two intentions the participants should have (1) the intention to move their hands within the 30‐s interval, (2) not to intentionally act at a certain point of time, i.e., not to have the intention to move their hands at a specific time. This forces the participants to become observers of themselves. Because they move their hands within the 30‐s interval, they have a preceding intention to act within this interval and this intention is causally effective, otherwise the participants would certainly not move their hands as expected. The non‐intended act, if it exists, regards the time within this 30‐s interval, when they move their hands. On the one hand the participants are expected, not to act intentionally, but only to observe their own decision behavior. On the other hand, the participants are expected to act intentionally.

On closer conceptual (philosophical) analysis there is a type of action that is intentionally controlled, i.e., the participant moves his or her hand within a specific 30‐s interval, and there is another type of action that should not be controlled intentionally by the participants, e.g., the participant moves his or her hand at the 17th second during the interval (if this is the moment of the concrete motor act of the participant). Since the intentionality of agency is always directed at types, never at tokens of acts, the conceptual analysis results in a contradiction: If one realizes one's intentions as an agent directed to some action type, one does this in the form of one or other specific action token. In the Libet experiment the participants realize their intentions in acting as expected, i.e., moving their hands within a 30‐s interval. The token—motor act at the 17th second—realizes the type—motor act within this 30‐s interval. What is unusual here is merely that the moment at which the participants move their hands should not be controlled intentionally. Therefore, they are forced to change roles from being agents to being observers of their own mental states (and report them: “I decided to move my hand, when the light dot was there”). The result of the experiment is likewise paradoxical: It goes without saying that the participants would not move their hands within the 30‐s interval without having the prior intention to do so (long before the RP evolves), but there is no prior intention for the more specific action type, e.g., moving the hand at the 17th second. The more comprising motor act is controlled intentionally, the more specific is not. This conceptual analysis shows that the widespread interpretation of the Libet results, which assumes that, in general, prior intention and the deliberation that determines the intention is irrelevant for human action, is an obvious non sequitur, since the relevance of prior intention is presupposed by the experimental design of the Libet study itself. But the conceptual analysis does not exclude so far that the more specific action type—moving the hand at the 17th second—is caused by RP alone.

To show that the RP alone does not suffice, or in other words, that deliberation and the resulting intentionality is in fact relevant for what we do, we must introduce an additional, deliberative element into the experimental design. This is done in our present study, which demonstrates that the deliberative element and the resulting intentionality is guiding the motor acts in a Libet‐like constellation. Our empirical findings and our interpretation thereof are compatible with both prevailing accounts in the contemporary philosophy of mind: (1) mind–body interactionism, which assumes that mental states and processes are not merely epiphenomena, but have a causal role in human agency, (2) mind–body‐identity theory, which assumes that mental states and processes are merely another description of neurophysiological ones, that mental states and processes are identical with the accompanying neurophysiological states and processes. The empirical findings of our experiment are metaphysically neutral in that sense, but they are not neutral regarding the widespread metaphysical interpretation of Libet's experiment as showing that the human self‐image is fundamentally wrong, that human action is not preceded by intentional decisions, but follows determinate brain processes that cannot be intentionally controlled by the agent.

### 1.2 Physiology of intended motor activation

Performing specific conscious self‐paced motor actions is preceded by specific electrical activity in the brain. The activity during voluntary hand movements has been studied by numerous groups (e.g., Gilden et al., 1966; Kornhuber & Deecke, 1964; Shibasaki et al., 1980; Toma et al., 2002; Vaughan et al., 1968) and shows a sequence of electrical components of different polarities primarily in the contralateral motor cortex. Initial components preceded the electromyographic activity of the wrist muscles by several 100 ms. Kornhuber and Deecke (1964, 1965) found that in self‐paced finger movements brain activity preceded the mechanical deflection of the finger by approximately 1000 ms. The authors introduced the term ‘Bereitschaftspotential’ or, English ‘RP’ for a characteristic, slowly increasing, negative potential followed by further electrical components, that were assigned to different origins in the brain. In a later series of experiments using the method of multichannel current source‐density (CSD) mapping with testing two types of brisk finger extensions, it was found that the early component of the RP started in the supplementary area, while the late component and the motor potential occurred as a contralateral preponderance of negativity (Cui et al., 1996). During bi‐manual sequential movements this aspect was studied further (Cui & Deecke, 2000) in another slow brain potential—the contingent negative variation (CNV; Walter et al., 1964). The complexity of the motor tasks affected the topography of the CNV so that its late component may stem from fronto‐central, central, centro‐parietal, parietal and parieto‐occipital areas, but meaning that the CNV and the RP was not identical.

Using fMRI‐constrained EEG dipole source analysis, Toma et al. (2002) demonstrated the existence of generators of movement‐related cortical potentials providing precise location and timing in right‐handed (RH) subjects during voluntary extension of the right index finger. Within bilateral SM1, activation of the precentral gyrus occurs bilaterally with similar strength from 1.2 s, followed by activation of the precentral bank from 0.5 s with contralateral predominance. Subsequently, the postcentral bank becomes active only on the contralateral side starting 0.1 s after movement (Toma et al., 2002). The times reported coincide with the well‐known components of the RP (e.g. Toma et al., 2002; −1.2 to −0.4 s), followed by the a much steeper Negative Slope (NS')‐component (starting at about −0.4 s) and finally the frontal peak of the motor potentials that was identified at about +0.1 s.

The experiments of Libet et al. (1983) were of relevance for both philosophy and physiology. The intention of the Libet‐experiment was to find a temporal correlation between brain activity patterns and the time at which subjects reported that the decision to act (move the hand or the finger) took place. For this approach subjects had to memorize the time of their decision. Time was represented by a clockwise rotating light point (2.56 s per rotation) on an oscilloscope screen providing a “clock position”. The result of this study was that the motor‐act‐initiating process starts at average 300 ms earlier than the memorized time of decision, raising critical questions about conscious initiation of motor acts.

The current study is based on two components, one of which is a go/no‐go (e.g. Heidlmayr et al., 2020) decision whereas the other, somewhat later, is a decision following a Stroop color‐word interference Stroop (1935), commonly used in experimental psychology. This type of double decision task requires an executive brain control and is employed in studies dealing with inhibition processes (e.g. Heidlmayr et al., 2020; Pires et al., 2014). The underlying complex cognitive functions of event‐related processes were found within four different time windows, with the first‐time window characterized by the go/no‐go task (N1/P2: 150–350 ms) and the other three intervals by the Stroop task (N2/P3: 200–400 ms, N400: 350–580 ms, and the late sustained potential [LSP]: 500–850 ms; Heidlmayr et al., 2020; see also figure 2 in Pires et al., 2014). According to Heidlmayr et al. (2020) the LSP can be divided into two different parts a negative deflection and a positive deflection that is also named late positive complex (Pires et al., 2014). P1, P2 and N1 were located at posterior electrode sites, whereas N2 and P3 were localized at fronto‐central electrodes. All these components are relatively separable and robust in time and location.

Both decisions, go/no‐go and Stroop color‐word interference, are considered as inhibition tasks (Heidlmayr et al., 2020; Larson et al., 2014), with go/no‐go representing motor inhibition and Stroop color‐word interference representing cognitive inhibition (Pires et al., 2014). Equal generators for the components are assumed. The corresponding generators for the N2 are thought to be in the anterior cingulate cortex (ACC), a structure believed to be responsible for conflict monitoring, the N400 as for the interference suppression and the LSP the conflict resolution (Heidlmayr et al., 2020).

Previously, we described experiments in a group of RH subjects and the above motor‐related areas only (Henz et al., 2015). In the current study both groups (RH) and left hand (LH) subjects were tested by the same procedures as described in the data analysis, based on our initial assumption of finding a difference in the similarity of activities in RH and LH. Thus, it is the aim of the current study to compare activity patterns in RH and LH obtained by a multi‐channel EEG analysis during a deliberation task resulting in a specific, voluntary, conscious, self‐paced motor action. In a color‐word interference Stroop task (Stroop, 1935) subjects had to decide whether (match‐, mismatch trials) or not (no‐go trials) to press button, depending on a randomly given information presented on a computer screen, without knowing the occurrence and the meaning of the next stimulus to which they had to respond. Thus, this conscious decision required a preceding mental process, localized in different cortical areas. By employing a multichannel EEG recording system, we will show process‐related activity patterns at discrete recording points above both hemispheres. Besides evaluation of the corresponding activity patterns obtained from discrete recording electrodes we employed the multi‐channel CSD analysis to define the spread of excitation above the whole cortex at discrete times. Moreover, the different trial types (match‐, mismatch trials, no‐go trials) were compared with respect to their occurrence of components in their time course.

## 2 MATERIALS AND METHODS

### 2.1 Ethical approval, subjects

Two groups of subjects consisting of a total of 28 RH and 21 left‐handed (LH) healthy students of the Ludwig‐Maximilians‐Universität München participated in this study. The study described has been carried out in accordance with The Code of Ethics of the World Medical Association (Declaration of Helsinki) for experiments involving humans. The study did not require a decision of the ethics committee because the examination involves a non‐invasive neurophysiological multi‐channel EEG measurement. The measurement is performed in a sitting position and is neither physiologically nor psychologically stressful. The subject collective comprises healthy students of the Ludwig‐Maximilians‐University of Munich (Munich, Germany). The local ethics committee—chaired by Professor Dr. W. Eisenmenger—of the medical faculty of Ludwig‐Maximilian—University of Munich was informed about the study (July 2, 2014). Each participant gave written informed consent prior to the start of the experiment and each was paid €35.00 for participation in a single 3‐h experimental session. Three RH and one LH were excluded for technical reasons. The initial criterion for exclusion of LH was reeducation from being LH to RH performance. The remaining group of RH (mean age ± SD: 22.9 ± 2.2 years; 18–27 years) consisted of 13 females and 12 males. The group of LH (mean age ± SD: 22.5 ± 2.5 years; 19–27 years) consisted of 11 females and 9 males. All LH and 12 RH subjects were tested according to the handedness incidence questionnaire in (Sattler, 1999). The questions were adapted to our requirements, with tests related to velocity of tracking of different types of mazes. In a further written test subjects were asked, e.g., with which hand scissors are held and used. All participants had normal or corrected‐to‐normal vision and had no history of neurological or psychiatric disorders.

### 2.2 Paradigm

The paradigm and the corresponding procedure have been described elsewhere (Henz et al., 2015) and are reported here only briefly. Electrophysiological signals preceding a voluntary motor act have been reported in many publications (Gilden et al., 1966; Kornhuber & Deecke, 1964; Shibasaki et al., 1980; Toma et al., 2002; Vaughan et al., 1968). If the motor act depends on a diversity of stimulus conditions, a deliberation process will precede the decision for a movement. In the current study, subjects had to press buttons with the index finger of the right or left hand, depending on the visual stimulus and on the subjects' handedness.

### 2.3 Stimulation protocol

During the experiment, participants sat comfortably in front of a standard 22 in. computer monitor (BenQ GL2250M, 16:9) at a distance of 80 cm. The subjects' hands rested on a table with the index fingers positioned on one button each. The screen showed regular checkerboard fields (24 in *x*‐direction and 20 in *y*‐direction) with a single field size of 1.43° × 0.96° respectively. During the experiment, participants had to fix their gaze on a gray fixation point in the center of the screen (0.32°).

#### 2.3.1 Visually evoked potential

Three types of stimulus‐reaction tasks were tested. In the initial series, visually evoked potentials (VEP) were recorded with the reversal of the checkerboard as stimulus. Repetitions (300) were given at an inter‐stimulus interval of 750 ms. An additional set of 300 VEPs (VEP‐text) were recorded, during which a text field indicating the name of a color (e.g. “red”) was shown for 300 ms, starting at the time of the reversal of the checkerboard. The size of the text field is provided below in the description of the third series of stimulus reaction tasks (see Section 2.3.3).

#### 2.3.2 Motor task

**FIGURE 1:**
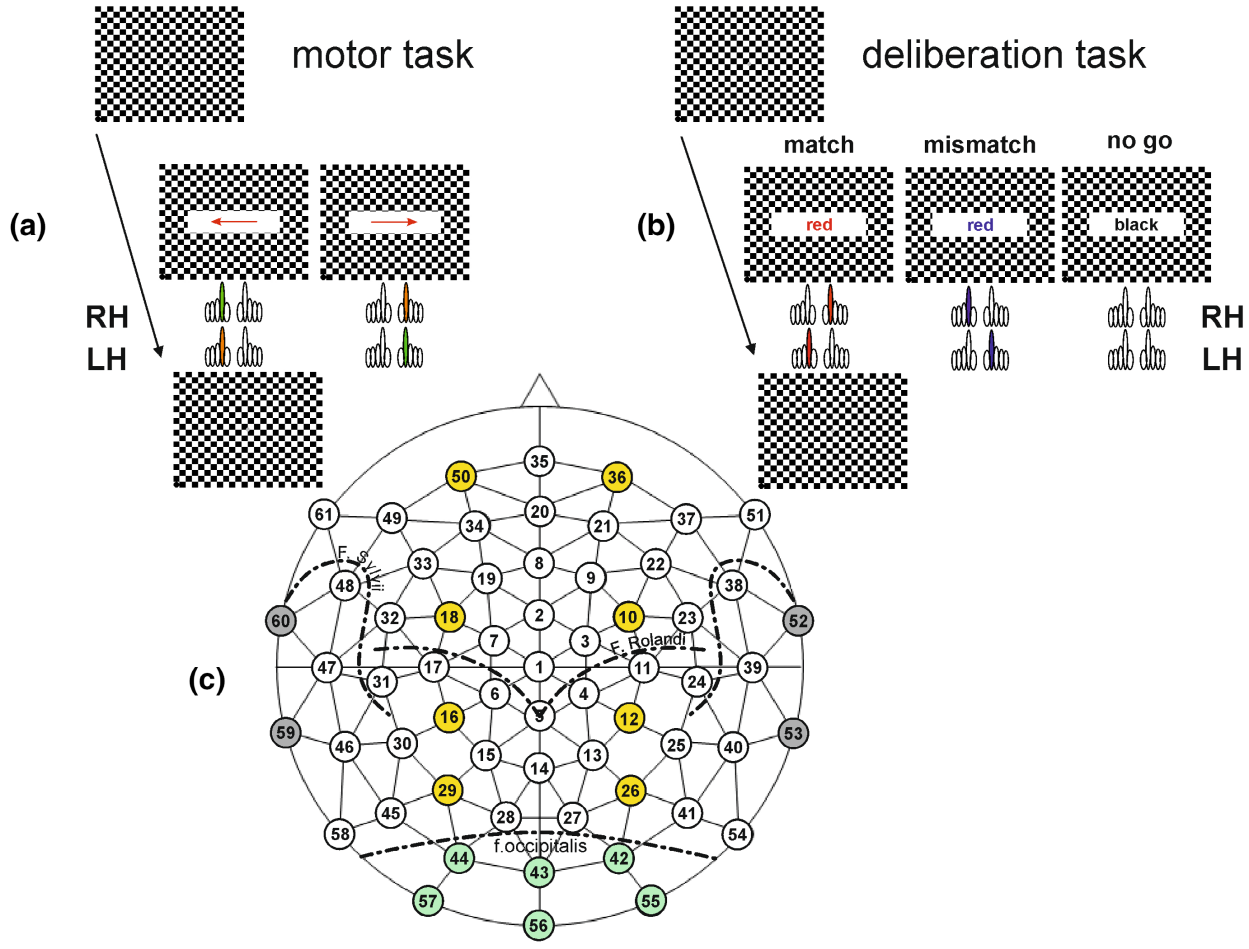
(a–c) Experimental design. (a) Subjects (right‐handed, *RH* or left‐handed, *LH*) had to perform initially a simple *motor task* (a) followed by the *deliberation task* (b). For both tasks they sat in front of a computer monitor showing a checker board. At the time of pattern reversal either an arrow (motor task) or a colored text (deliberation task) was shown for 300 ms. The inter‐stimulus interval was randomized (12–15 s). The checker board pattern reversal was recorded by an opto‐electronic sensor situated at the lower left corner of the screen (marked as black dot). (a) Subjects were informed about the direction of the arrow prior to the measurement of each of 50 trials. (b) During the deliberation tasks a text field with the written name of a color was shown at the time of pattern reversal for 300 ms. In match‐trials the name of the color coincided with the color of the text. Participants had to respond with the index finger of the dominant hand (meaning that RH had to respond with a keypress by the *right* index finger whereas LH had to respond with the *left* index finger [red]). Mismatch‐trials were characterized by inconsistency between the name of the color and the color of the text. In this case participants had to respond with the index finger of their non‐dominant hand. Hence, RH had to respond with a keypress by the *left* index finger whereas LH had to respond with a keypress by the *right* index finger (blue). *No‐go‐trials* were characterized by the word *‘black’* given in black color on the screen. During this type of trial subjects should not respond by any motor action. (c) Schematic representation of a 61‐electrode EEG‐cap with equidistant electrode positions. They are organized in concentric circles starting from the center electrode 1 in the middle of the head. The electrode positions come close to the 10–20 system (e.g. Jasper, 1958) and the revised version by (Jurcak et al., 2007). Electrodes with the numbers 52, 53, 59, and 60 are subdued and were not taken for analysis because of the problems due to the subjects’ ears. The responses of occipital electrodes marked by green dots are shown in Figure 2, whereas those of paracentral electrodes, marked by yellow dots, are shown in Figure 4.

In the second series, the subjects' reaction time was tested by recording the keypress of the index finger of the right and left hand (Figure 1a, motor task). For this test, each reversal of the checkerboard pattern was combined with the appearance for 300 ms of a right‐pointing arrow as stimulus, to which the RH had to respond with the right index finger (motor response to the right, MR), or a left‐pointing arrow for the left index finger (motor response to the left, ML). LH had to respond initially to a left‐pointing arrow as stimulus with the left index finger (ML), or, correspondingly, a right‐pointing arrow for the right index finger (MR). Subjects were aware of the direction of the arrow, during a block of 50 sequential repetitions in each direction at random inter‐stimulus intervals of 12–15 s. The size of the field in which the arrow was displayed was 66% of screen width (*x*‐direction: 316.8 mm; 21.6°), and 20% of screen height (*y*‐direction: 54 mm, 3.86°).

#### 2.3.3 Deliberation task

The third series consisted of the actual deliberation task, a type of color‐word Stroop‐task (Stroop, 1935) which consisted of 3 blocks of 100 trials each. The deliberation‐task conditions were presented at the same time as the reversal of the checkerboard pattern with a text field in the center of the screen (horizontal direction 66% of screen width, and in vertical direction 20% of screen height) as stimulus. Within the text field a color name was shown in color (Figure 1b, deliberation task).

In our original study (Henz et al., 2015), RH had to respond with the dominant hand. This was chosen explicitly for match‐trials so as to spread differences in behavior as well as in cortical responses. The current study therefore did not employ a counter‐balanced design. De facto, the reaction time of RH in the motor task has been shown to be independent of the hand used (Henz et al., 2015; Table 1). This design was retained to keep the current study comparable with (Henz et al., 2015).

**TABLE 1:** (contralateral, ipsilateral) Patterns of similarities expressed by correlation coefficients during contralateral match‐trials (column 1) and during contralateral mismatch‐trials (column 2), and during ipsilateral match‐trials (column 3) and during ipsilateral mismatch‐trials (column 4). Three different types of electrodes were correlated: (a) 6 pairs of marked yellow electrodes in Figure 1; (b) 26 pairs of unmarked electrodes in Figure 1; (c) 9 pairs of central electrodes. For example, the yellow electrode (#50 above left hemisphere) of a right‐handed subject in a contralateral match‐trial was correlated with a match‐trial of the yellow electrode (#36 above the right hemisphere) of a left handed subject. *r*‐Values, *p*‐values, lower 95% and upper 95% are given. For a Bonferroni correction of *p* at a level of <0.05 the *p* must be less than 0.000378

1: Match trials (contralateral)						2: Mismatch trials (contralateral)						3: Match trials (ipsilateral)						4: Mismatch trials (ipsilateral)					
RH: left hemisphere	LH: right hemisphere	*r*‐Value	*p*‐Value	Lower‐95%	Upper‐95%	RH: left hemisphere	LH: right hemisphere	*r*‐Value	*p*‐Value	Lower‐95%	Upper‐95%	RH: right hemisphere	LH: left hemisphere	*r*‐Value	*p*‐Value	Lower‐95%	Upper‐95%	RH: right hemisphere	LH: left hemisphere	*r*‐Value	*p*‐Value	Lower‐95%	Upper‐95%
Marked yellow in Figure 1c						Marked yellow in Figure 1c						Marked yellow in Figure 1c						Marked yellow in Figure 1c					
50	36	0.950	0.000	0.93	0.96	50	36	0.901	0.000	0.87	0.93	36	50	0.963	0.000	0.95	0.97	36	50	0.870	0.000	0.83	0.90
18	10	0.852	0.000	0.80	0.89	18	10	0.920	0.000	0.89	0.94	10	18	0.698	0.000	0.61	0.77	10	18	0.900	0.000	0.87	0.93
16	12	0.320	0.000	0.17	0.45	16	12	0.737	0.000	0.66	0.80	12	16	0.814	0.000	0.75	0.86	12	16	0.799	0.000	0.74	0.85
29	26	0.665	0.000	0.57	0.74	29	26	0.953	0.000	0.94	0.97	26	29	0.932	0.000	0.91	0.95	26	29	0.947	0.000	0.93	0.96
44	42	0.719	0.000	0.64	0.79	44	42	0.926	0.000	0.90	0.95	42	44	0.905	0.000	0.87	0.93	42	44	0.909	0.000	0.88	0.93
57	55	0.637	0.000	0.53	0.72	57	55	0.838	0.000	0.79	0.88	55	57	0.311	0.000	0.16	0.44	55	57	0.839	0.000	0.79	0.88
$\bar{x}$		0.690	0.000	0.61	0.76	$\bar{x}$		0.879	0.000	0.84	0.91	$\bar{x}$		0.771	0.000	0.71	0.82	$\bar{x}$		0.877	0.000	0.84	0.91
Unmarked in Figure 1c						Unmarked in Figure 1c						Unmarked in Figure 1c						Unmarked in Figure 1c					
34	21	0.569	0.0000	0.45	0.67	34	21	0.764	0.0000	0.69	0.82	21	34	−0.030	0.7070	−0.18	0.13	21	34	0.377	0.0000	0.24	0.50
49	37	0.804	0.0000	0.74	0.85	49	37	0.834	0.0000	0.78	0.88	37	49	0.384	0.0000	0.24	0.51	37	49	0.527	0.0000	0.40	0.63
61	51	0.724	0.0000	0.64	0.79	61	51	0.912	0.0000	0.88	0.93	51	61	0.654	0.0000	0.56	0.73	51	61	0.760	0.0000	0.69	0.82
19	9	0.805	0.0000	0.74	0.85	19	9	0.893	0.0000	0.86	0.92	9	19	0.689	0.0000	0.60	0.76	9	19	0.849	0.0000	0.80	0.89
33	22	0.636	0.0000	0.53	0.72	33	22	0.885	0.0000	0.85	0.91	22	33	0.617	0.0000	0.51	0.70	22	33	0.851	0.0000	0.80	0.89
48	38	0.621	0.0000	0.52	0.71	48	38	0.905	0.0000	0.87	0.93	38	48	0.889	0.0000	0.85	0.92	38	48	0.930	0.0000	0.91	0.95
32	23	0.713	0.0000	0.63	0.78	32	23	0.868	0.0000	0.82	0.90	23	32	0.856	0.0000	0.81	0.89	23	32	0.963	0.0000	0.95	0.97
7	3	0.844	0.0000	0.79	0.88	7	3	0.909	0.0000	0.88	0.93	3	7	0.720	0.0000	0.64	0.79	3	7	0.924	0.0000	0.90	0.94
17	11	0.849	0.0000	0.80	0.89	17	11	0.894	0.0000	0.86	0.92	11	17	0.740	0.0000	0.66	0.80	11	17	0.900	0.0000	0.87	0.93
31	24	0.867	0.0000	0.82	0.90	31	24	0.424	0.0000	0.29	0.54	24	31	0.205	0.0092	0.05	0.35	24	31	0.893	0.0000	0.86	0.92
47	39	0.687	0.0000	0.60	0.76	47	39	0.832	0.0000	0.78	0.87	39	47	0.954	0.0000	0.94	0.97	39	47	0.919	0.0000	0.89	0.94
6	4	−0.075	0.3437	−0.23	0.08	6	4	0.695	0.0000	0.60	0.77	4	6	0.651	0.0000	0.55	0.73	4	6	0.644	0.0000	0.54	0.73
15	13	0.605	0.0000	0.50	0.69	15	13	0.913	0.0000	0.88	0.94	13	15	0.838	0.0000	0.78	0.88	13	15	0.916	0.0000	0.89	0.94
30	25	0.425	0.0000	0.29	0.54	30	25	0.901	0.0000	0.87	0.93	25	30	0.858	0.0000	0.81	0.89	25	30	0.874	0.0000	0.83	0.91
46	40	0.618	0.0000	0.51	0.71	46	40	0.704	0.0000	0.62	0.77	40	46	0.493	0.0000	0.37	0.60	40	46	0.453	0.0000	0.32	0.57
28	27	0.863	0.0000	0.82	0.90	28	27	0.964	0.0000	0.95	0.97	27	28	0.890	0.0000	0.85	0.92	27	28	0.938	0.0000	0.92	0.95
45	41	0.310	0.0001	0.16	0.44	45	41	0.914	0.0000	0.88	0.94	41	45	0.797	0.0000	0.73	0.85	41	45	0.886	0.0000	0.85	0.92
58	54	0.774	0.0000	0.70	0.83	58	54	0.871	0.0000	0.83	0.90	54	58	0.600	0.0000	0.49	0.69	54	58	0.799	0.0000	0.73	0.85
$\bar{x}$		0.647	0.0191	0.56	0.72	$\bar{x}$		0.838	0.0000	0.79	0.88	$\bar{x}$		0.656	0.0398	0.57	0.73	$\bar{x}$		0.800	0.0000	0.74	0.85
Z‐electrodes^1^ in Figure 1c						Z‐electrodes^1^ in Figure 1c						Z‐electrodes^1^ in Figure 1c						Z electrodes^1^ in Figure 1c					
35	35	0.874	0.0000	0.83	0.91	35	35	0.857	0.0000	0.81	0.89	35	35	0.874	0.0000	0.83	0.91	35	35	0.857	0.0000	0.81	0.89
20	20	0.435	0.0000	0.30	0.55	20	20	0.667	0.0000	0.57	0.75	20	20	0.435	0.0000	0.30	0.55	20	20	0.667	0.0000	0.57	0.75
8	8	0.592	0.0000	0.48	0.68	8	8	0.800	0.0000	0.74	0.85	8	8	0.592	0.0000	0.48	0.68	8	8	0.800	0.0000	0.74	0.85
2	2	0.887	0.0000	0.85	0.92	2	2	0.932	0.0000	0.91	0.95	2	2	0.887	0.0000	0.85	0.92	2	2	0.932	0.0000	0.91	0.95
1	1	0.305	0.0001	0.16	0.44	1	1	0.759	0.0000	0.68	0.82	1	1	0.305	0.0001	0.16	0.44	1	1	0.759	0.0000	0.68	0.82
5	5	0.589	0.0000	0.48	0.68	5	5	0.758	0.0000	0.68	0.82	5	5	0.589	0.0000	0.48	0.68	5	5	0.758	0.0000	0.68	0.82
14	14	0.770	0.0000	0.70	0.83	14	14	0.932	0.0000	0.91	0.95	14	14	0.770	0.0000	0.70	0.83	14	14	0.932	0.0000	0.91	0.95
43	43	0.947	0.0000	0.93	0.96	43	43	0.949	0.0000	0.93	0.96	43	43	0.947	0.0000	0.93	0.96	43	43	0.949	0.0000	0.93	0.96
56	56	0.694	0.0000	0.60	0.77	56	56	0.938	0.0000	0.92	0.95	56	56	0.694	0.0000	0.60	0.77	56	56	0.938	0.0000	0.92	0.95
$\bar{x}$		0.677	0.0000	0.59	0.75	$\bar{x}$		0.844	0.0000	0.79	0.88	$\bar{x}$		0.677	0.0000	0.59	0.75	$\bar{x}$		0.844	0.0000	0.79	0.88

^1^ Z‐electrodes: Zero electrodes placed on the midline sagittal plane; 35: FPz, 20: AFz, 8: Fz, 1: Cz, 5: CPz, 14: Pz, 43: Oz, 56: Iz.

When the name of the color coincided with the print color shown, the trial is called *match*‐trial. RH had to press the right button with right index finger. If the name of the color did not coincide with the print color shown, RH had to press the left button (*mismatch*‐trial). Correspondingly, LH had to press the left button with the left index finger during *match*‐trials and the right button with the right index finger during *mismatch*‐trials. If the name of the color was “black” and was shown in black, no button was to be pressed (*no‐go* trial). The text fields were presented for 300 ms at randomized interstimulus intervals of 12–15 s with a probability of 1/3 for each trial type. The maximal response duration was set to 2000 ms after stimulus presentation.

### 2.4 Data recording

**FIGURE 2:**
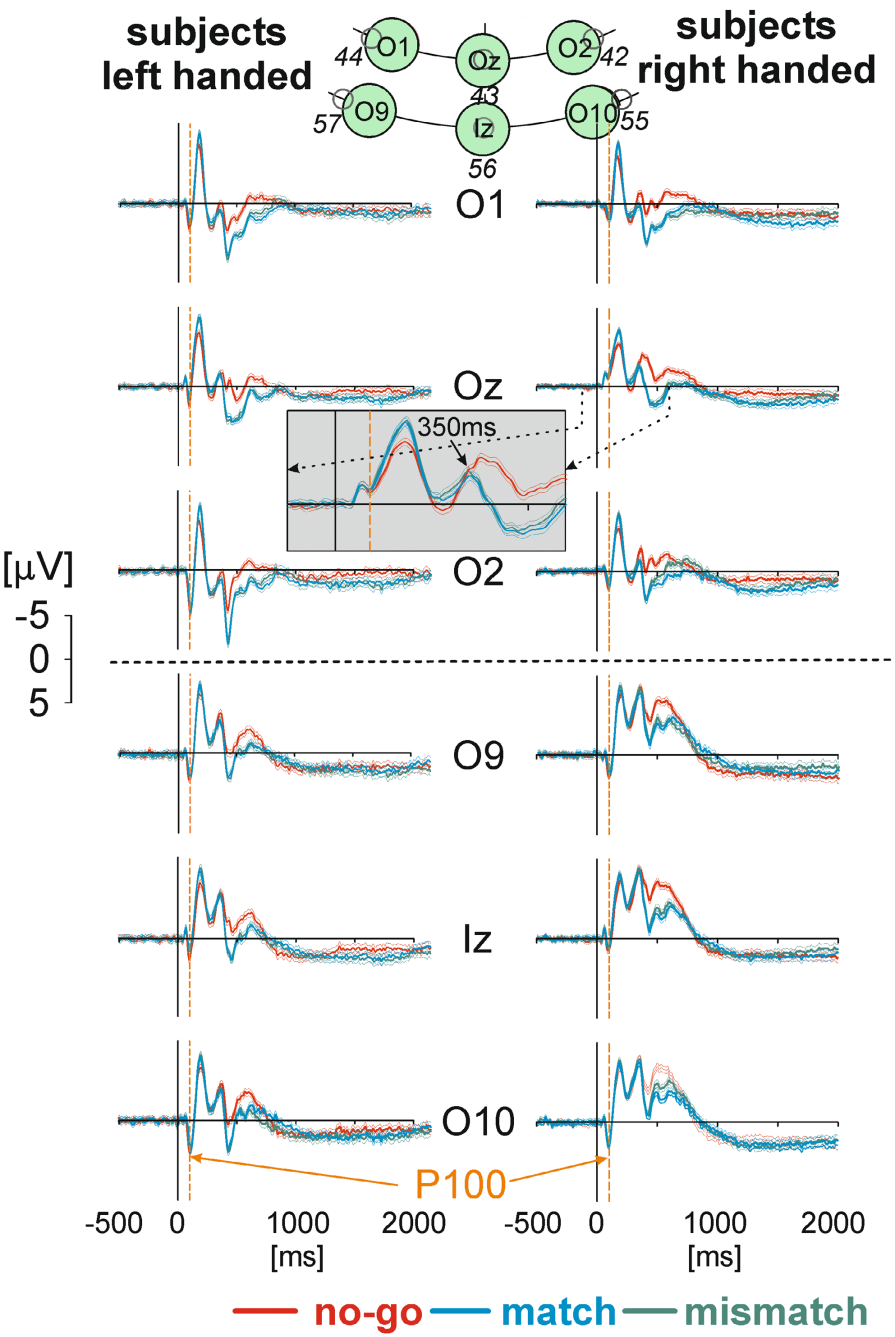
Comparison of occipital responses of left‐handed subjects (*LH*) with those of right‐handed subjects (*RH*). The responses of the occipital area are recorded from electrodes 42, 43, 44 (inner circle), 55, 56, 57 (outer circle, see Figure 1c), and correspond to the electrodes in vicinity of the 10–20 system (e.g. Jasper, 1958) O2, Oz, O1, O10, Iz, O9. All responses are stimulus‐aligned and cover the period from −500 to 2000 ms. Grand averages of EEG responses (thick solid lines) with 95% confidence limits of the group mean (thin solid lines) were obtained during the condition *not‐move (no‐go*‐trials, red), and *move* (*match‐* trials, blue, *mismatch*‐trials, green). All patterns are equally scaled from −5 to +5:V. The P100 time is marked by dashed orange lines. The shaded inset shows the responses of the Oz electrode of RH over an expanded time range (marked by dotted arrows). An arrow points to the time (350 ms) where the responses during no‐go‐trials start to be different to the responses during match‐and mismatch‐trials

During the whole session, the subjects' brain activity was recorded continuously via a computer‐assisted 64‐channel recording system (Electroencephalograph Neurofax EEG‐1200 pro; Nihon Kohden). Integrated in the Neurofax EEG‐1200 was a Sony video system (Sony EVI‐D70P) for simultaneous recording of the subject's face and facial muscle activity (e.g. frowning). Subjects wore an EEG‐cap (Montage No.10; Easycap GmbH) with 61 equidistant Ag/AgCl‐electrodes (distance: mean ± SD: 37 ± 3 mm, given at a head circumference of 58 cm). This results in electrode positions analyzed in this study on equidistant concentric circles around the central electrode (1) with increasing clockwise numbering (Figure 1c). Subdued electrode positions on the outer circle have been excluded from analysis, as no stable recordings could be made from these electrodes because of the subjects' ears. From two sets of electrodes, one set marked in green over the occipital lobe (see Figures 1 and 2) and from the yellow marked electrodes para‐central activities were recorded (see Figure 4). The green marked most caudal electrodes (el 42, 43, 44, 55, 56, 57, Figure 1c) are above the occipital lobe. They are situated very close to the electrode positions used clinically as the –10–20 system (e.g. Homan et al., 1987; Jasper, 1958). The electrode positions 42, 44, 55 and 57 are located slightly more laterally than those in the 10–20 system (top in Figure 2). The second group of electrodes, shown in yellow (Figure 1c), is para–centrally organized, starting from the occipital fissure to the region of the frontal lobe (see Figure 4).

The cap was mounted such that the center electrode matched the Cz = 1 position. The electrodes were backfilled with an electrolyte gel (Electro‐Gel; Electro‐Cap International Inc.) such that electrode impedances below 50 kΩ could be achieved. The reference electrode was fixed to the left earlobe, the ground electrode to the right earlobe. Electrophysiological data were sampled at 200 Hz, band pass‐ (0.01–70 Hz) filtered, and stored on the Neurofax EEG‐1200 pro computer for off‐line analysis. During recording a notch filter was activated only for the data display on the screen.

Stimulation and recording were controlled from a laptop computer (ASUS Pro, B53E), generating the checkerboard pattern and the temporal random sequence of the stimuli. The checkerboard pattern reversal was recorded by an opto‐electronic sensor (photo transistor of OPB 813 S) situated at the lower left corner of the screen (Figure 1a,b), thus providing an accurate time reference. For the press‐buttons, short‐way micro switches were used. The corresponding DC signals, obtained via a custom‐built interface, were fed to a microcontroller (Ethernet Atmega 32/644). The microcontroller evaluated the information of the checkerboard reversal, the trial type provided (match, mismatch, no‐go), the time between pattern reversals, the time of the subject's keypress (reaction time), whether the trial type corresponded to the performed key action, and fed them online as DC control signals to the Neurofax EEG‐1200 pro.

### 2.5 Data analysis

Trials with wrong responses (<2%) were excluded from further analysis, as they were artifact‐adhesive trials. The EEG waveforms were smoothed using a 0.01–40 Hz bandpass. To reduce the effect of blinks and eye‐movements on the EEG activity, we employed a specific eye‐movement detection and correction procedure using an artifact reduction tool (BESA Research analysis software, Version 5.3; BESA). With this software package, temporal sections of 500 ms preceding and 2000 ms following the stimulus could be cut out of the continuous data stream.

Generally, three types of time‐locked data are shown: (a) data related to the time at which the stimulus occurred (stimulus‐aligned); (b) data related to the time at which the key was pressed (key‐aligned) and (c) data related to maximal negativity, called N0, occurring prior to key press in the premotor areas.

Stimulus‐aligned data are available during the simple motor task and during match, mismatch and no‐go‐trials. Key‐aligned‐data are available during the simple motor task and during match‐ and mismatch‐trials whereas N0‐related potential‐data are available only during match and mismatch‐trials. Each trial taken into account—independent of stimulus type—was DC‐baseline‐corrected with the mean value obtained during a 400‐ms interval prior to stimulus onset. For this step a custom‐written analysis software package with corresponding algorithms based on the language for statistical computing R (Version 3.0.2, 2013‐09‐25; The R Foundation For Statistical Computing) was established in our laboratory. The data obtained from individual electrodes are presented as averaged or grand‐averaged responses (e.g. Figures 2 and 4).

The spatial distribution of these grand averages demonstrates the spread of excitation across the whole scalp at a certain time. The latter was documented by processing the activity of all electrodes using the CSD method provided by tools of BESA Research analysis software (Version 6.0; BESA). The CSD, and more appropriately current source‐ and sink‐density method is a useful tool for the analysis of membrane currents in laminated structures.

**FIGURE 3:**
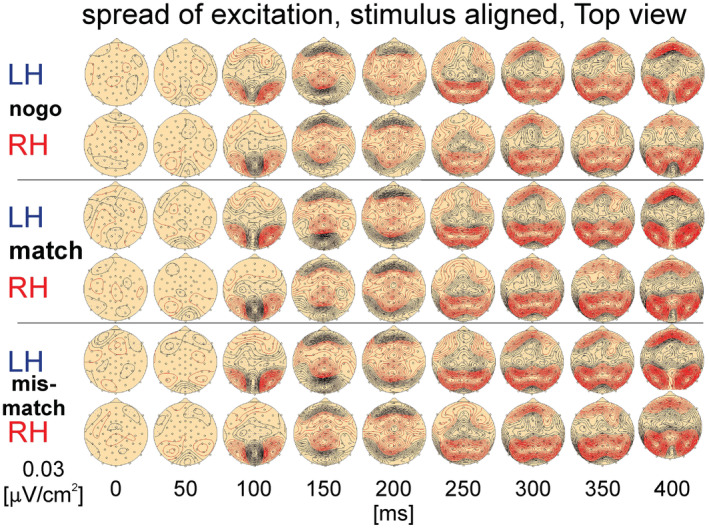
Spread of cortical excitation in right‐handed (*RH*) and left‐handed subjects (*LH*) during no‐go‐trials and move, match‐trials and mismatch‐trials. The results are stimulus‐aligned covering the period 0–400 ms. The data are presented in a top view Meridian Projection of a current source‐density mapping at a resolution of *[E]* = 0.03:V/cm^2^. Black contour lines represent sinks (surface negativity), red contour lines sources (surface positivity). The small circles represent the electrode positions.

The CSD *I*_m_—also called surface Laplacian operator—is a scalar quantity of dimensions of mA/cm^3^ and provides information about the distribution of current‐sinks (inward current) and current‐sources (outward current) in cells (see figure 4 in Kolb et al., 1997; Nicholson & Freeman, 1975; Nicholson & Llinas, 1975). The CSD generates a three‐dimensional current flow density vector ***J***_*x*,*y*,*z*_ that establishes the field potential Φ_*x*,*y*,*z*_. ***J*** and *I*_m_ are related through a divergence operation ∇ ***J*** = *I*_m_ (equation 3 in Nicholson & Freeman, 1975). In simpler terms, the CSD represents the second spatial derivative of the voltage distribution in tissue. A relation between current densities and surface EEG phenomena was investigated by Mitzdorf (1985). Positive surface potentials result from an activation of corresponding cells located in layer IV and V of the cortex producing a source on the distal dendrites. Negative surface potentials arise from an activation of inputs to the upper part of the cells producing a sink (Mitzdorf, 1985, figure 7). This was applied in this study and resulted in a corresponding spatial distribution of the EEG on the surface of the head, which is shown in a top view Meridian Projection of the CSD mapping at a resolution of [*E*] = 0.03 μV/cm^2^. Black contour lines represent sinks (negativities), red lines sources (positivities, see Figures 3 and 5).

### 2.6 Statistical analysis

**FIGURE 4:**
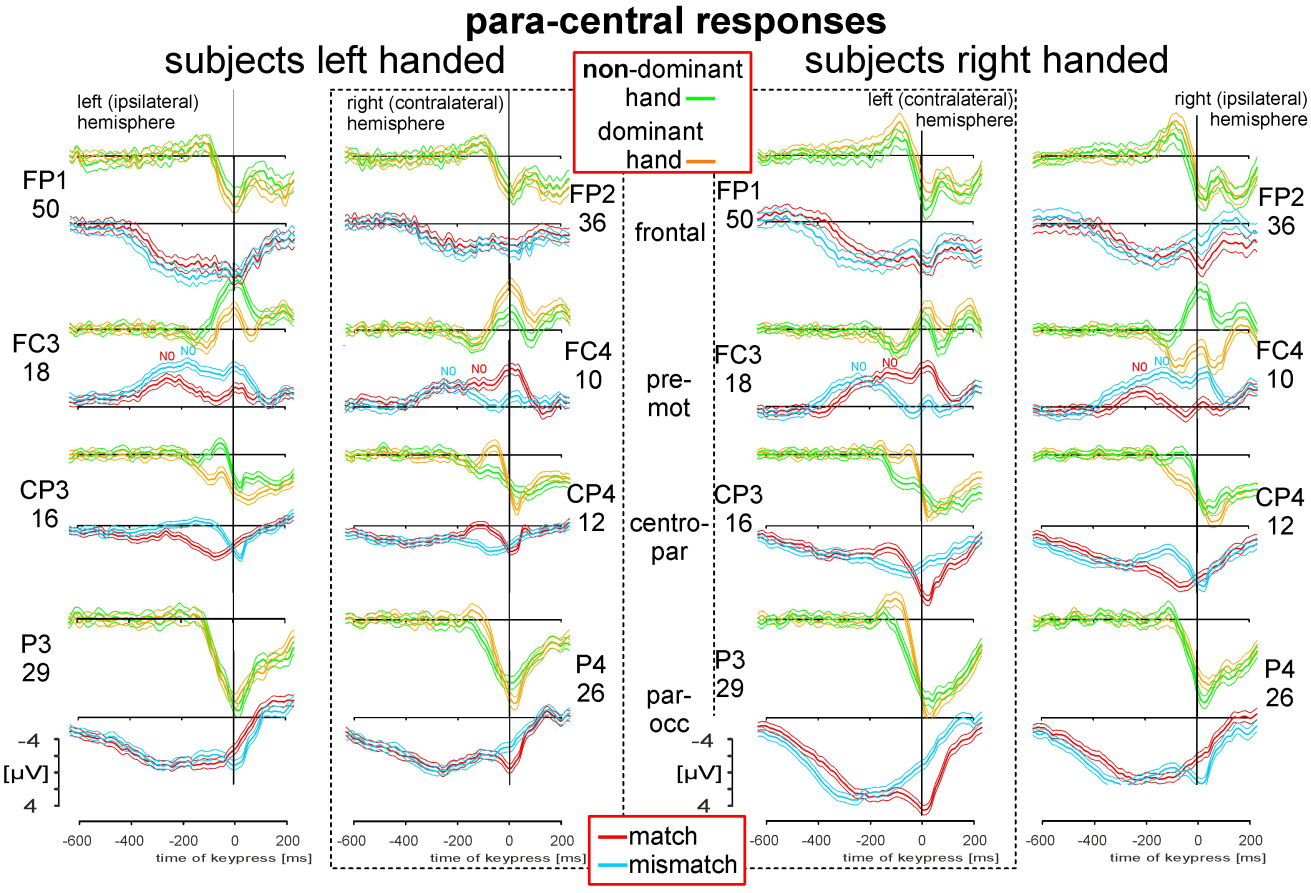
Para—central responses in left‐handed (*LH*, left two columns) and in right‐handed subjects (*RH*, right two columns) during simple motor trials with the dominant (orange traces) or non‐dominant (green traces) hand and during match‐trials (red traces) or mismatch‐trials (blue traces). The grand averaged EEG patterns (solid thick lines) with 95% confidence limits of the group mean (thin solid lines) were recorded from electrodes given in numbers (see Figure 1c) and in common abbreviations (see 10–20 system; e.g., Jurcak et al., 2007). The responses cover the period −600 to 200 ms, with the keypress at 0 ms. All patterns are scaled equally from −4 to +4:V. Small printed N0 components are shown for the premotor regions (el 18 (FC3) and 10FC4)). Electrodes located contralateral to the working hand and the same electrodes located ipsilateral to the non‐working hand are surrounded by dashed lines.

**FIGURE 5:**
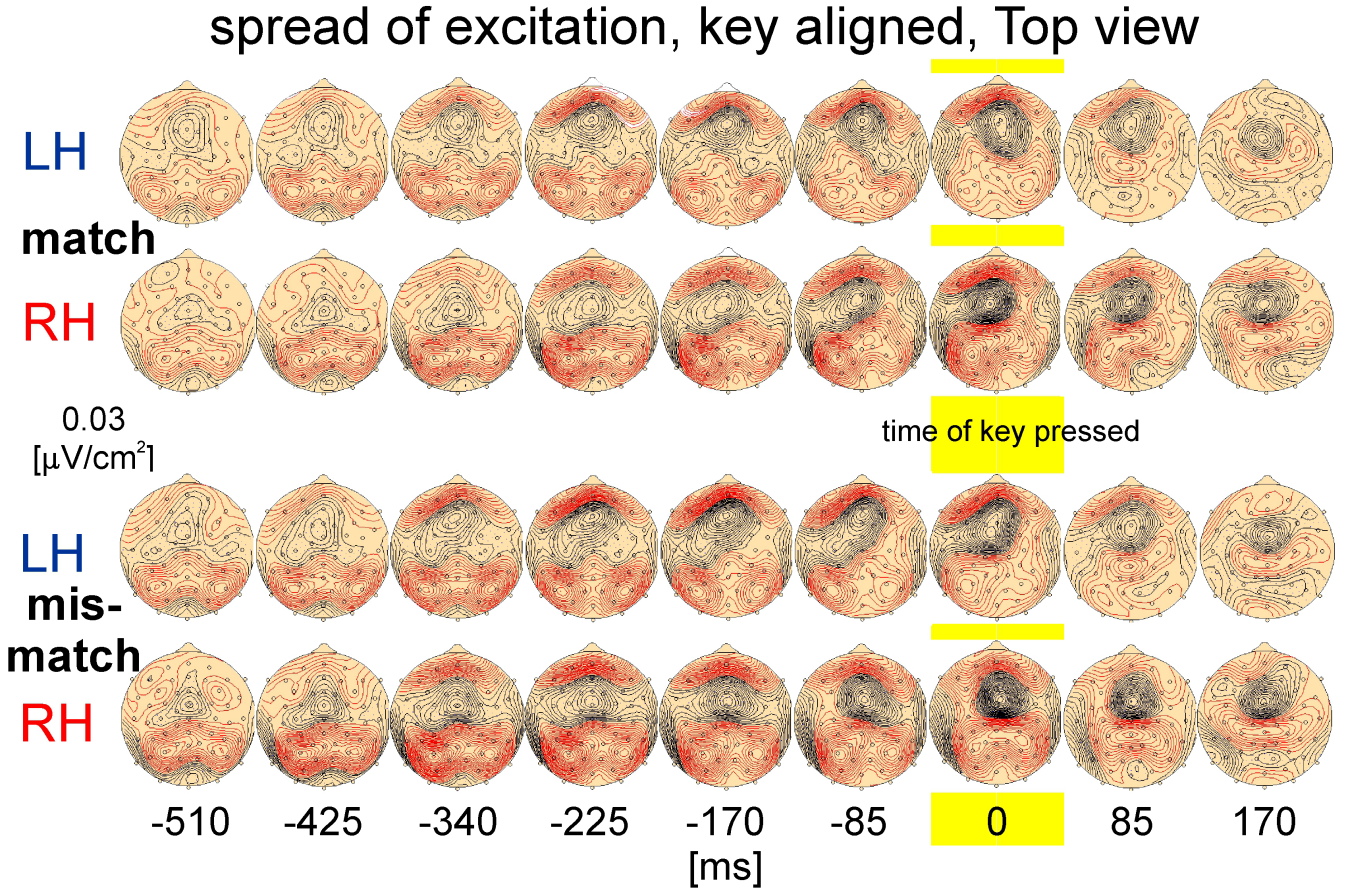
Spread of cortical excitation in right‐handed (*RH*) and left‐handed subjects (*LH*) during the deliberation tasks (match‐trials and mismatch trials). The patterns are aligned to the time of keypress (yellow marker, 0 ms) and covering the period −510 to 170 ms. The data are presented in a top view Meridian Projection of a current source density mapping at a resolution of [*E*] = 0.03:V/cm^2^. Black contour lines represent sinks (surface negativity), red contour lines mark sources (surface positivity). The small circles represent the electrode positions.

**FIGURE 6:**
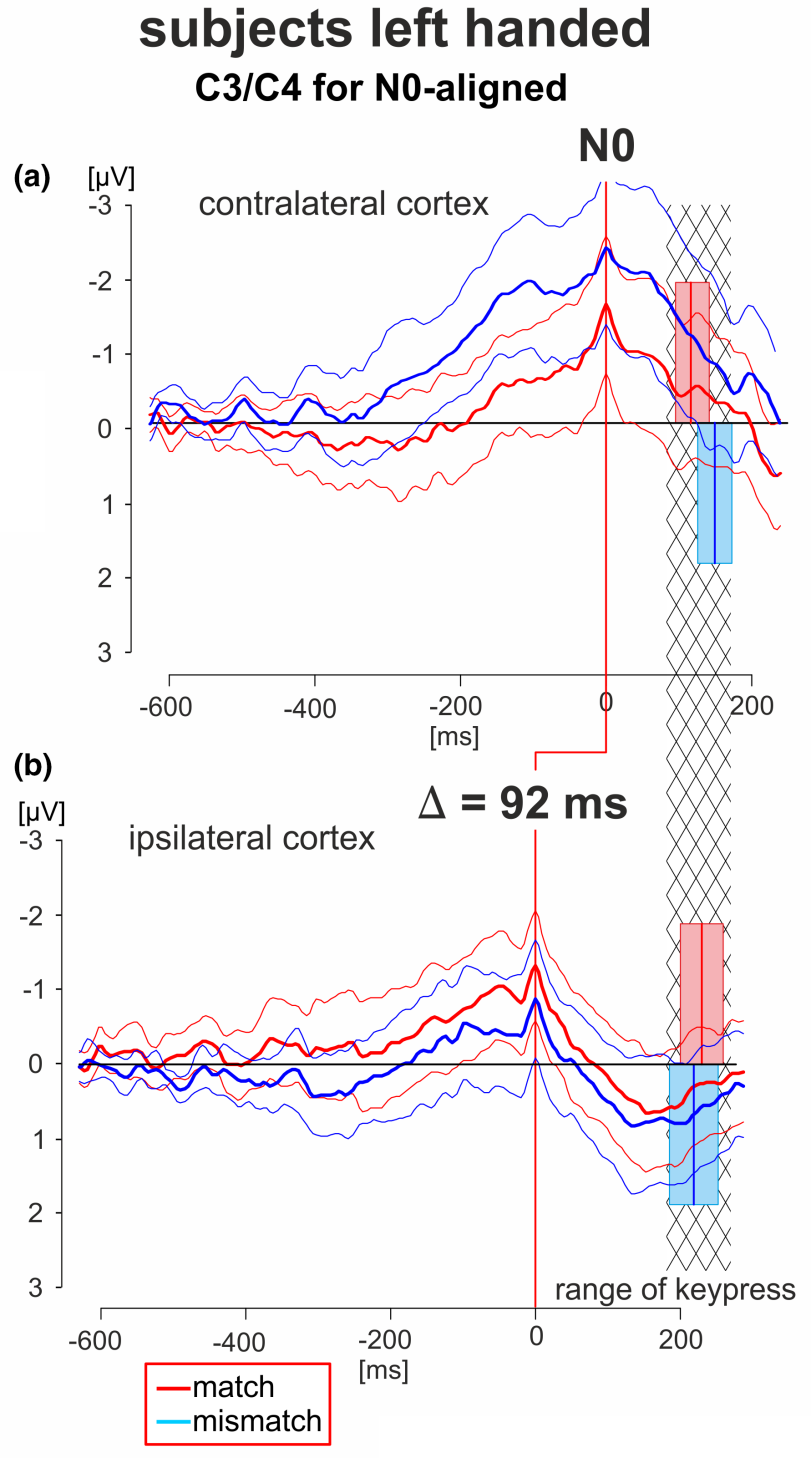
N0‐aligned EEG patterns obtained in left‐handed subjects from C3/C4 recording electrodes of the cortex areas contra‐ and ipsilateral to the working hand. Grand averaged EEG patterns (red lines during match‐trials, blue lines during mismatch‐trials, solid thick lines: averaged EEG patterns, solid thin lines: 95% confidence limits of the group mean). EEG patterns were obtained from the cortex contralateral (a) or ipsilateral (b) to the working finger, respectively, each of which are aligned to N0. The data cover the period from −600 ms before to 200 ms after N0, and are scaled equally (from −3 to +3:V). The red and blue blocks cover the time range (95% confidence limits, thin solid lines) of the times of keypress (mean value: solid thick line) during match and mismatch‐trials, respectively. EEG patterns in (a) and (b) are also N0‐aligned such that the contra‐ and ipsilateral responses are within statistical limits (95% confidence limits), indicated by the hatched areas.

Analysis time for stimulus‐aligned data was the 500 ms interval preceding the stimulus onset to 2000 ms after (Figures 2 and 3), for key‐aligned data 600 ms preceding keypress to 200 ms after (Figures 4 and 5), for N0‐potential‐aligned data 600 ms prior to and 200 ms after keypress (Figure 6).

N0 is the maximal negativity of the slowly increasing activity prior to keypress in the precentral region component. The detection of the N0 peak is a semi‐automatic process, meaning that the range of the putative occurrence has to be selected by the user whereas the program calculates the corresponding latency and amplitude automatically. The onset of this increasing activity was calculated by an automatic regression model describing the data with two different linear splines (see Figure 6). The time window starting from the stimulus onset and terminating at the N0 peak was divided into two intervals at any time point within the time window. Linear regression analyses were performed for both intervals and the onset of the increasing negativity was determined in a least‐squares setting as that point in time at which the residues of both regressions resulted in a minimal value (Meindl et al., 2012). The minimal length of each interval for the regression analyses was set to 50 ms. For determining whether the maximal potential correlated with either stimulus onset or keypress, variances of the durations of the intervals stimulus onset to N0 and N0 to keypress were analyzed using an adaptation of the Kepner‐Randles test (Kutz et al., 2003). For details see Henz et al. (2015). All further statistical comparisons (mean values, standard error of the mean [SEM], 95% confidence limits of the mean) were obtained using our laboratory‐developed software package based on the language for Statistical Computing R. For the group analysis, these parameters were treated by appropriate statistical tests, such as unpaired, two‐tailed *t*‐tests. *p* < 0.05 was assumed to be significant. Due to the high variability of the EEG signals the various components could not be detected in individual trials but only from the averaged response of each individual electrode position per subject by the semi‐automatic peak detection analysis mentioned above.

A correlation analysis of the group averages (Snedecor & Cochran, 1989) was used to quantify a given similarity between corresponding patterns of RH and LH. In RH the activity pattern obtained from a given electrode position (e.g. el 18 = FC3 above *contra*lateral [*left*] cortex) was correlated with the activity pattern in LH obtained from the corresponding electrode position (e.g. el 10 = FC4 above [*right*] cortex). Resulting correlation coefficients have been calculated during matched and mismatched conditions, and, with respect to the moving hand, from the contralateral and from the ipsilateral cortexes (Figure 4; Table 1).

## 3 RESULTS

The aim of this study was to compare the activity patterns of 25 young and healthy RH subjects with those of 20 LH subjects. The subjects' specific handedness was first tested with regard to gross and fine motor skills. For this purpose, the test of Sattler (1999), especially developed for left‐handers, was used, whereby a maximum of 20 points could be achieved. LH subjects reached 16.8 ± 2.4 points for performing the task with the dominant left hand and 0.8 ± 1.0 points when performing the task with the right hand.

Right‐handed were subjects from our previous study (Henz et al., 2015). Twelve of the 25 participants were subsequently recruited to take part in the Sattler test of handedness. They reached 18.9 ± 1.2 points for performing the task tested with the dominant, right hand and 0.3 ± 0.5 points if the task was tested with the left hand.

The activity patterns were obtained by recording from high density EEG recordings during VEPs and during simple motor tasks, both as controls for reaction time. The main task consisted of a deliberation task leading to a motor act. The deliberation task was characterized by pressing one of two buttons depending on the stimulus on the screen with either the right or the left index finger, based on a color‐word Stroop task (Stroop, 1935). Matched trials required the RH to respond with the right index finger and the LH with the left index finger. Correspondingly mismatched trials required the response of RH with the left index finger and that of LH with the right index finger. Depending on specific information, given on the screen, subjects were not to press any button, so called no‐go trials.

### 3.1 Decisions of subjects for the trial types

The activities over occipital areas from *discrete* electrodes (el 42, 43, and 44 from the inner cycle and 55, 56, 57 from the outer cycle in Figure 1c, shown by green color; and O2, Oz, O1, O10, Iz, O9, Figure 2, green) from both types of subjects (RH and LH) were studied during all types of trials. Data are presented stimulus‐aligned. The decision consisted of two steps: In the first decision RH and LH decided either to move (match‐trials, mismatch‐trials, both in blue in Figure 2) or not‐to‐move (no‐go‐trials, red, Figure 2). The 95% confidence limits are added to the averaged responses. This can be seen qualitatively in the gray‐shaded inset on an expanded time scale taken from RH from the Oz electrode. As can be seen match‐, mismatch‐, no‐go‐trials do not differ significantly during the first 300 ms after the stimulus (Figure 2). The separation between match‐ and mismatch‐ versus no‐go‐trials obtained from Oz (el43) was calculated via a z‐transformation and appeared in RH at 369.2 ± 17.2 ms, and in LH: 355.0 ± 15.5 ms. This is the time at which the activities over the occipital lobe during no‐go‐trials (in red) exceed the 95% confidence limit in both groups of subjects. The result represents the start of subject's initial decision move versus not‐to‐move. The second decision is related to match‐ and mismatch‐trials only and can better be shown in key‐aligned data (see Figure 4). The positivity P100 component in VEPs established in clinics is marked by a dashed orange line (Figure 2).

### 3.2 Comparison of the spread of excitation in the different trial types

The stimulus‐aligned neuronal activity during no‐go‐, match‐ and mismatch‐trials is shown by the spread of excitation across the whole cortex in a top view Meridian Projection. The spread of excitation, based on the current source density (CSD), for RH and LH is displayed at discrete times (Figure 3), covering a comparable time range, as shown in the inset in Figure 2 (0–400 ms after stimulus).

The *P100* component represents a sink that is sharply circumscribed and can be discerned clearly during all trial types and in both groups of subjects. The component is larger in RH, particularly during match‐trials. Maximal values were found within a triangle given by the occipital electrodes 27, 43, 28 (corresponding for the 10–20 system: PO1, Oz, PO2, e.g. Jasper, 1958) on both sides of the parieto‐occipital sulcus (see Figure 1c). In its lateral vicinity the appearance of the corresponding sources can be seen, particularly in LH on the right parieto‐occipital region of the cortex. Frontal sources are clearly smaller at that time but larger in LH in all trial types.

In the period between *150 and 200 ms* frontally and occipitally situated sink activities are established, with accompanying sources in between. At *150 ms* and during match‐trials both RH and LH establish small sinks in the right parieto‐temporal region. At *200 ms* the source split in two or three centrally located components.

In the period between *250 and 300 ms* the strong frontally and occipitally situated sink activity observed at 150 and 200 ms is clearly reduced. In addition, a medially expanding minor sink appears with sources primarily over the parietal and occipital and less pronounced over frontal regions.

In the period between *350 and 400 ms* the medially located sink activities enlarge in both lateral directions, with several sink peaks, particularly at *400 ms* and in match‐ and mismatch‐trials. The similarity of the activities in match‐ and mismatch‐trials for both RH and LH reflect the result shown in Figure 2. The above mentioned medially expanded sink is clearly smaller during no‐go‐trials, particular in RH compared with match‐ and mismatch‐trials.

### 3.3 Second decision of subjects in keypress‐trials on discrete potential patterns

The subjects' second decision can be derived from the comparison between the activities over para‐central regions during simple motor tasks and during the deliberation tasks for both groups of subjects (Figure 4). The activities of LH and RH were recorded from corresponding discrete electrodes (marked yellow, Figure 1c) located over the subjects contralateral and ipsilateral hemispheres with respect to the working hand. During simple motor tasks all averaged potentials are key‐aligned and color‐coded: For RH, orange traces (Figure 4) indicate the activity obtained with the right index finger (dominant hand) and green traces with the left index finger (non‐dominant hand). For LH these colors are reversed (orange for the use of left index finger and green for the right index finger). The responses during simple motor tasks have to be compared with respect to the occurrence of the responses during match‐ and mismatch‐trials. During deliberation tasks, responses in red are related for match‐trials, and blue for mismatch‐trials. The 95% confidence limits are added to all averaged responses. The columns surrounded by broken lines represent the corresponding contralateral hemispheres for LH and RH (Figure 4).

The corresponding waveforms of the group averages of RH and LH were tested for symmetry using the correlation test of Snedecor and Cochran (1989). Correlation coefficients, probabilities and 95% lower and upper limits of coefficients are given in Table 1. This applies to the corresponding contralateral hemispheres (with respect to the handedness) as well as to ipsilateral hemispheres. For example, a correlation between patterns obtained from the contralateral premotor areas of *RH (el 18)* and *LH (el 10)* during *match‐trials* (red in Figure 4) results in *r*_match, contra, marked yellow_ = 0.852 (Table 1, 1st column, line 2). For all para‐central electrodes (marked in yellow Figure 1c) and during match‐trials the overall *r*_match, contra, marked yellow, all_ = 0.690 (Table 1, 1st column). Correspondingly, during *mismatch‐trials* (blue in Figure 4) the correlation between patterns of RH (el 18) and LH (el 10) results in *r*_mismatch, contra, marked yellow_ = 0.920 (Table 1, 2nd column, line 2). For all para‐central electrodes (marked in yellow, Figure 1c) and during mismatch‐trials the overall *r*_match, contra, marked yellow, all_ = 0.879 (Table 1, 2nd column). The 18 unmarked, lateral electrodes (Table 1, 1st column) result during match‐trials in *r*_match, contra, unmarked, all_ = 0.647 and during mismatch‐trials in *r*_mismatch, contra, unmarked, all_ = 0.838 (Table 1, 2nd column). The correlation of the nine Zero electrodes placed on the midline sagittal plane calculated between RH and LH results during match‐trials *r*_match, zero electrodes, all_ = 0.677 (Table 1, 1st column) and during mismatch‐trials *r*_mismatch, central, all_ = 0.844 (Table 1, 2nd column). The correlation coefficients obtained from the corresponding *ipsilateral* hemispheres of RH and LH were larger for match‐trials compared with *contralateral* match‐trials in (Table 1, 3rd column). The same holds true when comparing those during mismatch‐ and match‐trials from the corresponding ipsilateral hemispheres of RH and LH (Table 1, 4th column). In short, it can be stated that stimulus‐aligned activity patterns of RH and LH are fairly mirror symmetrical. This holds true for matched‐trials as well as for mismatched‐trials, obtained from contralateral as well as from the ipsilateral hemispheres. The sequence of individual components in a given activity pattern obtained from an individual electrode has been described previously in detail for different motor areas in RH (Henz et al., 2015). These components were also found for LH and only the slowly increasing negativity, termed N0, was evaluated in this study for the premotor and motor areas.

The N0 component was assumed to represent the end of the deliberation process (Henz et al., 2015). Comparison of the averaged activities during *key*‐alignment match and mismatch trials above premotor and motor areas (Table 2A, yellow and reddish highlighted values) indicates that N0 terminates significantly earlier on the cortex *ipsi*lateral to the working hand (mean: ~60 ms, range: [41–74 ms]; Student's *t*‐test, *p* < 0.05, Bonferroni corrected) than on the *contra*lateral side. Comparison of the averaged activity during *stimulus*‐aligned match and mismatch trials shows a significant difference only for RH for the electrodes of the right cortex (C4, FC4, FCC2, Table 2B, yellow highlighted values). This indicates that during mismatch‐trials the cortical activity decreases significantly earlier than during match‐trials, although the entire development of the match process is still incomplete and thus occurs ~60 ms closer to key press (Table 2). These results indicate that N0 is clearly related to the keypress, based on the decision which hand has to be used. The components in frontal, centro‐parietal and parieto‐occipital regions show positive potentials at different times prior to keypress for RH and LH (Figure 4).

**TABLE 2:** N0‐latencies and amplitudes are taken from right‐handed and left‐handed subjects. All values are presented as group MEAN ± SEM [ms]. *n*: Number of subjects taken into account. (A) N0 calculated during key‐aligned trials. The N0 were obtained during match trials (red in Figure 4) and mismatch trials (blue in Figure 4) of right‐handed and left‐handed subjects: 17, 18, 7 (C3, FC3, FCC1) and electrodes: 11, 10, 3 (C4, FC4, FCC2). Corresponding mean values between match and mismatch trials are highlighted by yellow respectively reddish color, expressing significance (*p* < 0.05, Bonferroni corrected). (B) N0 calculated during stimulus‐aligned trials. The table is constructed as described for A. Mean values between match and mismatch trials were significantly different (*p* < 0,05, Bonferroni corrected) in right‐handed subjects only and are highlighted by yellow color. The values of right‐hand subjects were reported (Henz et al., 2015) and are repeated here for comparison. (C) Reaction times of right‐handed and left‐handed subjects during match‐ and mismatch‐trials. Reaction times differ significantly between trials in each group (Student's *t*‐test, *p* < 0.001, Bonferroni corrected)

A	N0 during key‐aligned			
	Right handed subjects *n* = 25		Left handed subjects *n* = 20	
Cortex	Electrode	(ms)	Electrode	(ms)
Match (red)				
Contralateral to the working hand	17, C3	−85.4 ± 7.3	11, C4	−111.3 ± 4.2
	18, FC3	−111.2 ± 7.9	10, FC4	−98.0 ± 5.6
	7, FCC1	−142.4 ± 9.6	3, FCC2	−108.8 ± 6.1
Mean		−113.0 ± 8.3		−106.0 ± 5.3
Ipsilateral to the working hand	11, C4	−186.6 ± 7.9	17, C3	−205.0 ± 5.3
	10, FC4	−167.4 ± 8.7	18, FC3	−157.8 ± 5.2
	3, FCC2	−200.4 ± 11.5	7, FCC1	−166.0 ± 10.2
Mean		−184.8 ± 9.4		−176.3 ± 6.9
Mismatch (blue)				
Ipsilateral to the working hand	17, C3	−173.6 ± 10.4	11, C4	−206.5 ± 8.2
	18, FC3	−164.4 ± 11.0	10, FC4	−163.8 ± 7.4
	7, FCC1	−157.4 ± 10.4	3, FCC2	−171.0 ± 6.3
Mean		−165.1 ± 10.6		−180.4 ± 7.3
Contralateral to the working hand	11, C4	−88.8 ± 8.1	17, C3	−104.0 ± 6.0
	10, FC4	−138.6 ± 10.0	18, FC3	−142.3 ± 8.1
	3, FCC2	−125.8 ± 9.3	7, FCC1	−160.3 ± 6.3
Mean		−117.7 ± 9.1		−135.5 ± 6.8

B	N0 during stimulus‐aligned			
	Right handed subjects *n* = 25		Left handed subjects *n* = 20	
Cortex	Electrode	(ms)	Electrode	(ms)
Match (red)				
Contralateral to the working hand	17, C3	623.0 ± 24.2	11. C4	723.9 ± 19.6
	18, FC3	595.2 ± 17.4	10. FC4	676.1 ± 23.9
	7, FCC1	564.0 ± 21.44	3. FCC2	665.4 ± 25.8
Mean		594.1 ± 20.8		700.0 ± 23.1
Ipsilateral to the working hand	11, C4	519.8 ± 20.5	17. C3	569.1 ± 23.4
	10, FC4	539.0 ± 18.7	18. FC3	616.4 ± 26.0
	3, FCC2	506.0 ± 24.4	7. FCC1	608.1 ± 20.6
Mean		521.6 ± 21.2		597.9 ± 23.3
Mismatch (blue)				
Ipsilateral to the working hand	17, C3	596.5 ± 22.2	11. C4	610.1 ± 23.4
	18, FC3	605.7 ± 22.2	10. FC4	652.8 ± 25.9
	7, FCC1	612.7 ± 23.47	3. FCC2	645.6 ± 26.2
Mean		601.1 ± 22.2		636.2 ± 25.2
Contralateral to the working hand	11, C4	681.3 ± 20.9	17. C3	712.6 ± 22.9
	10, FC4	631.5 ± 18.3	18. FC3	674.3 ± 26.2
	3, FCC2	644.3 ± 23.5	7. FCC1	656.3 ± 27.4
Mean		652.4 ± 20.9		681.1 ± 25.5

C	Reaction times (ms)	
	Right handed subjects	Left handed subjects
Match trials	706.4 ± 20.5	774.1 ± 24.1
Mismatch‐trials	770.1 ± 19.1	816.6 ± 26.0
Difference	63.7	42.5

### 3.4 Tracking the second decision of subjects in keypress‐trials via the spread of excitation activity

The comparison of the activity patterns in keypress trials of RH and LH during match‐ and mismatch‐trials is shown in top view Meridian Projections of CSD maps (Figure 5). It shows the spread of excitation in a time range from −510 ms prior to 0 ms (keypress, marked yellow) and finally to 170 ms. During match‐ and mismatch trials and up to −340 ms the distribution of sources and sinks of RH and LH is approximately symmetrical with respect to an imaginary vertical center line. Over the occipital lobe decreasing small sources can be discriminated and which are presumably due to the visual input of the stimulus as has been shown in Figure 3. Moving from the occipital lobe to the frontal lobe two increasing bilateral sources arise, followed by a centrally located single sink and ending in a frontally situated broad single source. The latter remains until 85 ms after keypress with maximal values around the time of keypress.

During *mismatch‐trials* in RH (working with the left index finger) an initial, contralateral, right‐parietal sink, starting from −85 ms prior to keypress, separating from the single centrally located sink, with large values and expanding to the right hemisphere. Correspondingly and during the same time range, LH show during mismatch‐trials, working with the right index finger, established at −85 ms a strong, contralateral sink of the anterior, fronto parietal cortex, expanding over the left hemisphere.

This pattern is very similar to that in RH during match‐trials (right index finger is working) with large values in the centrally located sink, expanding to the left hemisphere. In match‐trials in LH (left index finger is working) the shift of a sink is less pronounced with a maximal value at −85 ms. For all trials and for both groups of subjects, absolute maximal values occur at the time of keypress. Thereafter all amplitudes of sources and sinks gradually decrease. In summary, sources and sinks spread from occipital regions, with sources of large positivities, expanding medially located sinks, that shift—as expected—to the corresponding hemispheres of the working finger.

### 3.5 EEG patterns with the N0 component as the end of the deliberation task

As has been shown in our previous study in RH only, the component N0 in the motor region represents the end of the deliberation process, followed by a sequence of P1‐N1‐P2 potentials (Henz et al., 2015). In premotor regions the N0 is the peak of a continuously increasing negative potential. Because of the large variability of N0, the onset was calculated by a regression model describing the data with two different linear splines (Meindl et al., 2012). By applying an appropriate two tailed‐test procedure (Kepler‐Randles Test, Kutz et al., 2003) it has been shown that the N0 component is related significantly to keypress but not to stimulus onset. Figure 4 in this study shows the N0 components during match‐ and mismatch‐trials over premotor hemispheres. Corresponding components may be positive, example, over the frontal cortex, centro‐parietal cortex, and parieto‐occipital cortex. The analysis of the occurrence of the N0‐aligned data in the contralateral und ipsilateral cortexes (with respect to the handedness) was performed in this study for LH during match‐ and mismatch‐trials in the C3/C4 motor range and is shown in Figure 6. The data are N0‐aligned with a reference time point at keypress. Because of the N0‐alignment the corresponding keypress events are scattered resulting in red blocks for match‐trials and blue blocks for mismatch‐trials representing the 95% confidence limits. The temporal overlap of both blocks is marked by the hatched area (Figure 6). Independently of the trial type, the N0 component occurred 92 ms earlier in the ipsilateral cortex than in the contralateral cortex. This is within the range (95 ms) of that in RH (Henz et al., 2015).

## 4 DISCUSSION

The aim of this study was to compare the activity of RH with LH subjects. Both groups had to perform three different tasks. The clinical VEP was assessed (as a control) to determine whether the visual stimulation elicited typical responses. The reaction times and responses of the second task (simple motor task) were tested for comparison to those of the deliberation task. The deliberation task was based on the color‐word Stroop (1935) task during which subjects knew neither when the stimulus occurred nor how they had to respond. In contrast to the deliberation tasks, during the simple motor task subjects were informed in advance about the meaning of the stimulus but not about the time of its occurrence.

### 4.1 Groups of subjects

Two groups of subjects participated in the current study, one being RH, the other LH. To verify their handedness subjects had to perform the handedness incidence questionnaire (Sattler, 1999). The percentages of our handedness tested subjects (see Section 3), particularly the LH group, were reliable as re‐education from initially LH to RH was a criterion excluding participation in the LH group. Furthermore, due to early psychological reasons reeducation in Germany was performed more frequently up to 1970 but reduced thereafter. The dominance of the corresponding cortical hemisphere is not necessarily related to hands only, because a dominance is also found in legs, eyes, and ears. Thus, the percentage of LH versus RH is very variable with values up to 10%; however, the percentage is probably larger (up to 20%, Sattler J.B. personal communication) if subjects were born later than 1970 and not reeducated.

### 4.2 Double‐decision task

#### 4.2.1 First decision

Both groups of subjects had to perform a double decision task. As can be seen qualitatively in Figure 2 (gray‐shaded inset at on expanded time scale taken from the Oz electrode of RH) and quantitatively from a statistical analysis (*z*‐Transformation) the separation (time after stimulus onset) between match‐, mismatch‐, and no‐go‐trials was 369.2 ± 17.2 ms for RH, and 355.0 ± 15.5 ms for LH and can be interpreted as the time for the decision to move or not to move. Experiments dealing with this aspect have been described in the literature (e.g. Haggard, 2008; Haggard & Eimer, 1999; Trevena & Miller, 2010). Trevena and Miller found in their experiment 1 for three experimental conditions (“Sometimes move, moved”; “Sometimes move, did not move”; “Always move, moved”) a movement‐preceding, slowly increasing, small negativity at electrode Cz prior to stimulus. Because they could not find any difference in these preceding negativities during the condition “Sometimes move, move” versus the condition “Sometimes move, not to move” they concluded that this negativity is not related to an expected movement preparation (Gomes, 2010; Trevena & Miller, 2010). A dissonant opinion is that of Brunia (2003) who stated that it is comparable to the CNV (Walter et al., 1964) and thus would be a movement‐preceding negativity like the Readyness Potential (RP; e.g. Deecke et al., 1969). However, the CNV may also be present in the absence of movement (Trevena & Miller, 2010). In the present experiments the situation differs the above studies (Brunia, 2003; Trevena & Miller, 2010; Walter et al., 1964). As reported previously (Henz et al., 2015) there was no pre‐stimulus activity in either voltage‐direction. Because methods and paradigm are identical in the current study, no activity change in the pre‐stimulus period should be expected. The main reason for not having a stimulus‐preceding negativity or an expectancy, such as the CNV, may be derived from the fact that our subjects did not know how to respond to the upcoming stimulus (moving with either hand or do not move at all).

### 4.3 Spread of excitation of the different trial types

The potentials presented in Figure 2 were obtained from individual electrodes (green in Figure 1c) and show the discrete activity of the cortex below. To show the dynamic spread of excitation the CSD method was employed. This technique has been employed for cortical tissues in animals and was described by Nicholson and Freeman (1975) initially, and Nicholson and Llinas (1975) and was subsequently employed in cats (Kolb et al., 1997; Mitzdorf, 1985). The CSD is proportional to the sum of the second derivatives of the potential field (MacKay, 1984) and shows the localization of the electrical activities more precisely than the raw potential distribution, and thus is far more sensitive to local sources, both tangentially and in depth (Cui et al., 1996, 1999; Nunez et al., 1994). The technical basis for human EEG processing is part of the dipole source analysis (Brain Electric Source analysis, BESA, e.g. Scherg, 1990; Scherg and Berg, 1991; Scherg et al., 2019). This method allows the evaluation of the topography of current sources and sinks within the brain.

For the first decision, a sink (surface negativities, black contour lines) occurred in the occipital region only approximately at 50 ms, accompanied by sources (surface positivities, red contour lines), bilaterally in neighboring areas but with no qualitative difference in RH and LH and with no difference for the type of trial (Figure 3). At 100 ms these patterns increased their strengths of both, sources and sinks, and spread to medial regions with maximal amplitudes of sinks between 150 and 200 ms. Up to 250 ms the previous patterns reduced their strengths in occipital regions whereas frontal sinks were established. The patterns obtained later than 300 ms are characterized by frontally and parietally located sources with a medially located band of sinks.

The amplitudes of the voltage potentials of all types of trials within the expanded section (350 ms, Figure 2) are almost similar, but changing after this section. The qualitative distribution of the CSD patterns is approximately equal for RH and LH and thus, are independent of the trial types (Figure 3).

#### 4.3.1 Second decision

The subjects' second decision can be seen during the color‐word Stroop task (Stroop, 1935). The responses were triggered by the subjects' pressure on the corresponding keys and were characterized by movements only (key‐aligned results). These responses were characterized by slowly negative increasing cortical potentials in the central region (Figure 4, FC3/FC4) and slowly positive increasing cortical potentials in pre‐frontal (Figure 4, FP1/FP2) and parietal regions (Figure 4, CP3/CP4 and P3/P4).

Slowly surface increasing negative cortical deviations in freely moving rats with chronically implanted electrodes were reported by Caspers (1963). In humans, similar increasing negative responses preceding volitional finger movements were reported initially by Kornhuber and Deecke (1964, 1965). These authors introduced the term Bereitschafts Potential (German, BP) or RP. Besides these purely motor‐driven potentials Benjamin Libet (Libet et al., 1982), modified the approach in the sense that subjects had to memorize the time at which they consciously decided to perform an intentional motor act. These results led Libet to suggest that free will is an illusion. In similar experiments (e.g. Haggard & Eimer, 1999; Keller & Heckhausen, 1990) subjects had to memorize the time of their decision with the aim of finding a relationship between the mental perception of the intended movement and the measured electrophysiological signals prior to the cognition. This, however, differs from both our previous study (Henz et al., 2015) and the current study, in which subjects had to make the decision to move depending on the color of the color‐word as initially introduced by Stroop (1935).

As mentioned above, there was no activity prior to the appearance of the color word on the screen. This contrasts with the study of Trevena and Miller (2010), as shown earlier (Henz et al., 2015). The latter study analyzed primarily premotor and motor areas in RH, and demonstrated homogeneous patterns above the contralateral and ipsilateral hemispheres. Prior to keypress different components (P1‐N1) have been observed during the required volitional finger movements and with the P1 related to the pre‐motor positivity (e.g. Deecke et al., 1976). The late P2 in Henz et al. (2015) was assumed to be a feedback signal. Vaughan et al. (1968) reported a four‐component complex (N1‐P1‐N2‐P2). A slowly increasing negativity has been observed and reported in the literature under different experimental conditions and was described first as the BP by Kornhuber and Deecke (1964, 1965) and later by Shibasaki et al. (1980) and Barrett et al. (1986). Cui et al. (1996) studied the onset of the BP and the underlying structures involved during right finger extensions of different loads (low load task, high load task). With the method of CSD they found that the small BP 1 in the fronto‐central midline and its corresponding current sink appeared as early as 2.3 s before EMG onset. Subsequently these authors documented the onset of BP 2 at 0.97–0.41 s (0.69 ± 0.2 s; Cui et al., 1999). The BP 2 was of higher density compared to BP 1, and, moreover, lateralized toward the contralateral hemisphere. Toma et al. (2002) analyzed movement related cortical potentials using 56 scalp electrodes and EEG dipole source analysis combined with fMRI data. With this technique they were able to demonstrate the fronto‐central areas described by Cui et al. (1996, 1999). Furthermore, after six dipoles (three on each side of the corresponding primary sensory‐motor cortex) were seeded at activated spots obtained by the fMRI, the dipole orientations were fixed. The activation of the precentral gyrus had similar strengths from at −1.2 s, followed by a contralateral preponderance at −0.5 s. Thus, the post central bank becomes active on the contralateral side 0.1 s after movement.

The slowly increasing surface negativity preceding the keypress as shown in Henz et al. (2015) terminated at an early component termed N0. It was interpreted as the end of the deliberation process. The appearance of the N0 components depended on the working hand and on the trial type such that N0 on the hemisphere contralateral to the working hand (e.g. #17 = C3) obtained during match trials was found at −85.4 ± 7.3 ms prior to keypress (*t* = 0 ms). The N0 obtained during mismatch trials at the same electrode—now ipsilateral to the working hand and contralateral to the non‐working hand—was found at −173. 6 ± 10.4 ms. This means that the N0 value over the corresponding cortex of the non‐working hand peaks significantly earlier (see table 2 and figure 3 in Henz et al., 2015).

In the current study the corresponding paracentral responses of LH (left two columns) and RH (right two columns) are shown (Figure 4). Simple motor tasks were used as controls for the timing of the deliberation tasks. The results obtained during the deliberation task, i.e., during the color‐word Stroop task (1935), are shown in red (match‐trials) and blue (mismatch‐trials). The N0 component can be detected primarily within the paracentral premotor areas (FC3, FC4, Figure 4). As was the case for the subjects in our previous study (Henz et al., 2015), the N0 evoked over the corresponding cortex of the non‐working hand appears earlier than the N0 obtained over the corresponding cortex of the working hand. This holds true for both, LH and RH in the current study. It is assumed that the N0 components in the other responses may exist, but they are difficult to detect. As shown in Figure 4, the individual components obtained over the corresponding cortex areas were similar for RH and LH.

Because subjects did not know which hand was to be used to press the corresponding key in the upcoming color‐word Stroop trial (1935), both hands are prepared initially. This type of task is accepted as a conflicting mental process due to the difficulties of evaluating both the color of the text on the screen and its meaning. This is not related to the subjects' handedness, as there is no difference between LH and RH and no difference in the pure simple motor tasks, during which subjects know exactly the type of the upcoming movement (but not the time). The conflict situation during Stroop color‐word interference has been described in detail in the literature. In conflict trials both motor cortices are activated as has been shown by DeSoto et al. (2001). Motor conflict in Stroop tasks have been studied by Szucs et al. (2009) and show a robust congruency effect that appeared in the amplitude of congruent versus incongruent event‐related potentials (ERPs) between 330 and 400 ms, and which may be related to the activity of the ACC. Stimulus–aligned data as shown in Figure 2 during the first decision of our subjects look very similar to the LSP of the congruent and incongruent ERP over the period 350–700 ms (Liotti et al., 2000, Figure 3 “Manual”, with data obtained during a Stroop color‐word approach). Corresponding results have been found for the same period (350 ms; figure 2B,C in Larson et al., 2014) as distinct, slow potential conflict monitoring processes. In a review by Heidlmayr et al. (2020) temporal and anatomical aspects during Stroop executive control processes were discussed for conflict monitoring (200 ms), interference suppression (N400 ms), and conflict resolution by the LSP (600 ms). These values are comparable with those reported by Larson et al. (2014): The Flanker N2 (300 ms) is followed by the conflict slow potential (at 450 ms) with a shape of the averaged waveform depending on congruent or incongruent trials, recorded over the ACC, representing conflict monitoring processes. In inhibition studies performed with high‐resolution ERP for three periods (200 ms after stimulus onset, 200–400 ms, and 400–800 ms) the inhibition was found primarily at N400 and N450 at the central electrode site (i.e. Cz and CPz, Pires et al., 2014).

The above‐mentioned authors used stimulus‐aligned paradigms only whereas the N0 components in the current study were clearly related to the time of keypress. Correspondingly a relation between the above‐mentioned times cannot be derived directly from the different times of N0. As shown previously (Henz et al., 2015), the N0 was attributed to the end of the deliberation. If, however, the Stroop‐color‐word conflict is taken into account, N0 evoked by the non‐working hand may also be the beginning of the conflict processing. The time remaining between N0 and the keypress is relatively short, but it seems to be sufficient before and/or during the execution of the motor act. At the current stage of research the spread of excitation expressed by different emergences and shifts of sources and sinks, as shown in Figure 5, cannot be attributed to individual conflict processes.

An early inhibitory process has been assumed for RH (Henz et al., 2015). This was based on studies of DeSoto et al. (2001) and Szucs et al. (2009) that provide evidence that, as long as the deliberation process is incomplete, an initial bilateral activation of both cortical hand areas has to be accepted. The N0 peak above the corresponding cortical area of the non‐working hand has to be canceled actively.

The method of N0‐alignment established and applied for RH in Henz et al. (2015) has now been extended to LH as well (Figure 6). The times between the contralateral and ipsilateral C3 and C4 were within the same time range (RH: Δ = 95 ms; LH: Δ = 92 ms), indicating that there was no qualitative difference between RH and LH. In agreement with Carter et al. (1998), Gehring and Knight (2000), DeSoto et al. (2001) the cortical region primarily responsible for processing the resulting conflict seems to be the ACC, even if additional cortical regions have to be taken into account.

Based on the quantitative similarity of the activities and of the locations in the LH and RH our hypothesis of providing functional differences in these groups is not supported but yields some insights into processing a two‐step decision in a deliberation task.

## AUTHOR CONTRIBUTIONS

Florian P. Kolb, Dieter F. Kutz, Walter Hürster, Julian Nida‐Ruemelin: project conceptualization; Florian P. Kolb, Julian Nida‐Ruemelin, Dieter F. Kutz: funding acquisition and project administration; Jana Werner, Sonja Schönecker, Dieter F. Kutz, Florian P. Kolb: data collection and analysis; Dieter F. Kutz and Florian P. Kolb: data revision, conceptualization of analysis; Florian P. Kolb: current source density analysis, and writing of the first version of the manuscript; Florian P. Kolb, Dieter F. Kutz, Jana Werner, Sonja Schönecker, Walter Hürster, Julian Nida‐Ruemelin: writing, review and editing; All authors contributed to manuscript revision and read and approved the submitted version. All authors have read and agreed to the published version of the manuscript.

## FUNDING INFORMATION

This study was supported by the Munich Center for Neurosciences—Brain and Mind, Ludwig‐Maximilians‐University, Grosshaderner Str.2, 82152 Martinsried, Germany, Project: JNR‐2011.

## CONFLICT OF INTEREST

The authors declare that the research was conducted in the absence of any commercial or financial relationships that could be construed as a potential conflict of interest.

## ETHICS STATEMENT

The study described has been carried out in accordance with The Code of Ethics of the World Medical Association (Declaration of Helsinki) for experiments involving humans. The study did not require a decision of the ethics committee because the examination involves a non‐invasive neurophysiological multi‐channel EEG measurement. The measurement is performed in a sitting position and is neither physiologically nor psychologically stressful. The subject collective comprises healthy students of the Ludwig‐Maximilians‐University of Munich (Munich, Germany). The local ethics committee—chaired by Professor Dr. W. Eisenmenger—of the medical faculty of Ludwig‐Maximilian—University of Munich was informed about the study (July 2, 2014). Each participant gave written informed consent prior to the start of the experiment and each was paid €35.00 for participation in a single 3‐h experimental session.

## References

[phy215522-bib-0001] Barrett, G. , Shibasaki, H. , & Neshige, R. (1986). Cortical potentials preceding voluntary movement: Evidence for three periods of preparation in man. Electroencephalography and Clinical Neurophysiology, 63, 327–339. 2419090 10.1016/0013-4694(86)90017-9

[phy215522-bib-0002] Brunia, C. M. H. (2003). CNV and SPN: Indices of anticipatory behavior. In M. Jahanshahi & M. Hallett (Eds.), The bereitschaftspotential: Movement‐related cortical potentials (p. 21). Kluwer Academic/Plenum Publishers.

[phy215522-bib-0003] Carter, C. S. , Braver, T. S. , Barch, D. M. , Botvinick, M. M. , Noll, D. , & Cohen, J. D. (1998). Anterior cingulate cortex, error detection, and the online monitoring of performance. Science, 280, 747–749. 9563953 10.1126/science.280.5364.747

[phy215522-bib-0004] Caspers, H. (1963). Relation of steady potential shifts in the cortex to the wakefullness‐sleep spectrum. In M. A. Brazier (Ed.), Studies of the brain functions (pp. 177–123). Universtiy of California Press.

[phy215522-bib-0005] Cui, R. Q. , & Deecke, L. (2000). High resolution DC‐EEG of the Bereitschaftspotential preceding anatomically congruent versus spatially congruent bimanual finger movements. Brain Topography, 12, 117–127. 10.1023/a:1023414328616 10642011

[phy215522-bib-0006] Cui, R. Q. , Huter, D. , Lang, W. , & Deecke, L. (1999). Neuroimage of voluntary movement: Topography of the Bereitschaftspotential, a 64‐channel DC current source density study. Neuroimage, 9, 124–134. 9918734 10.1006/nimg.1998.0388

[phy215522-bib-0007] Cui, R. Q. , Huter, D. , Lang, W. , Lindinger, G. , Beisteiner, R. , & Deecke, L. (1996). Multichannel DC current source density mapping of the Bereitschaftspotential in the supplementary and primary motor area preceding differently loaded movements. Brain Topography, 9, 83–94.

[phy215522-bib-0008] Deecke, L. , Grözinger, B. , & Kornhuber, H. H. (1976). Voluntary finger movement in man: Cerebral potentials and theory. Biological Cybernetics, 23, 99–119. 949512 10.1007/BF00336013

[phy215522-bib-0009] Deecke, L. , Scheid, P. , & Kornhuber, H. H. (1969). Distribution of readiness potential, pre‐motion positivity, and motor potential of the human cerebral cortex preceding voluntary finger movements. Experimental Brain Research, 7, 158–168. 5799432 10.1007/BF00235441

[phy215522-bib-0010] DeSoto, M. C. , Fabiani, M. , Geary, D. C. , & Gratton, G. (2001). When in doubt, do it both ways: Brain evidence of the simultaneous activation of conflicting motor responses in a spatial stroop task. Journal of Cognitive Neuroscience, 13, 523–536. 11388924 10.1162/08989290152001934

[phy215522-bib-0011] Fried, I. , Mukamel, R. , & Kreiman, G. (2011). Internally generated preactivation of single neurons in human medial frontal cortex predicts volition. Neuron, 69(3), 15–562. 10.1016/j.neuron.2010.11.045 PMC3052770 21315264

[phy215522-bib-0012] Gehring, W. J. , & Knight, R. T. (2000). Prefrontal‐cingulate interactions in action monitoring. Nature Neuroscience, 3, 516–520. 10769394 10.1038/74899

[phy215522-bib-0013] Gilden, L. , Vaughan, H. G., Jr. , & Costa, L. D. (1966). Summated human EEG potentials with voluntary movement. Electroencephalography and Clinical Neurophysiology, 20, 433–438. 4143682 10.1016/0013-4694(66)90100-3

[phy215522-bib-0014] Gomes, G. (2010). Preparing to move and deciding not to move. Consciousness and Cognition, 19, 457–459. 20079665 10.1016/j.concog.2009.10.008

[phy215522-bib-0015] Haggard, P. (2008). Human volition: Towards a neuroscience of will. Nature Reviews Neuroscience, 9, 934–946. 19020512 10.1038/nrn2497

[phy215522-bib-0016] Haggard, P. , & Eimer, M. (1999). On the relation between brain potentials and the awareness of voluntary movements. Experimental Brain Research, 126, 128–133. 10333013 10.1007/s002210050722

[phy215522-bib-0017] Heidlmayr, K. , Kihlstedt, M. , & Isel, F. (2020). A review on the electroencephalography markers of Stroop executive control processes. Brain and Cognition, 146, 10. 10.1016/j.bandc.2020.105637 33217721

[phy215522-bib-0018] Henz, S. , Kutz, D. F. , Werner, J. , Hurster, W. , Kolb, F. P. , & Nida‐Ruemelin, J. (2015). Stimulus‐dependent deliberation process leading to a specific motor action demonstrated via a multi‐channel EEG analysis. Frontiers in Human Neuroscience, 9, 355. 26190987 10.3389/fnhum.2015.00355 PMC4488757

[phy215522-bib-0019] Homan, R. W. , Herman, J. , & Purdy, P. (1987). Cerebral location of international 10–20 system electrode placement. Electroencephalography and Clinical Neurophysiology, 66, 376–382. 2435517 10.1016/0013-4694(87)90206-9

[phy215522-bib-0020] Jasper, H. H. (1958). Report of the committee on methods of clinical examination in electroencephalography. Electroencephalography and Clinical Neurophysiology, 10, 370–375.

[phy215522-bib-0021] Keller, I. , & Heckhausen, H. (1990). Readiness potentials preceding spontaneous motor acts: Voluntary vs. involuntary control. Electroencephalography and Clinical Neurophysiology, 76, 351–361. 1699728 10.1016/0013-4694(90)90036-j

[phy215522-bib-0022] Kolb, F. P. , Arnold, G. , Lerch, R. , Straka, H. , & Büttner‐Ennever, J. A. (1997). Spatial distribution of field potential profiles in the cat cerebellar cortex evoked by peripheral and central inputs. Neuroscience, 81, 1155–1181. 9330375 10.1016/s0306-4522(97)00255-8

[phy215522-bib-0023] Kornhuber, H. H. , & Deecke, L. (1964). Hirnpotentialänderungen beim Menschen vor und nach Willkürbewegungen, dargestellt mit Magnetbandspeicherung und Rückwärtsanalyse. Pflügers Archiv European Journal of Physiology, 218, 52.

[phy215522-bib-0024] Kornhuber, H. H. , & Deecke, L. (1965). Hirnpotentialänderungen bei Willkürbewegungen und passiven Bewegungen des Menschen: Bereitschaftspotential und reafferente Potentiale. Pflügers Archiv European Journal of Physiology, 284, 1–17. 14341490

[phy215522-bib-0025] Kutz, D. F. , Fattori, P. , Gamberini, M. , Breveglieri, R. , & Galletti, C. (2003). Early‐ and late‐responding cells to saccadic eye movements in the cortical area V6A of macaque monkey. Experimental Brain Research, 149, 83–95. 12592506 10.1007/s00221-002-1337-9

[phy215522-bib-0026] Larson, M. L. , Clayson, P. E. , & Clawson, A. (2014). Making sense of all the conflict: A theoretical review and critique of conflict‐related ERPs. International Journal of Psychophysiology, 93(3), 283–297. 24950132 10.1016/j.ijpsycho.2014.06.007

[phy215522-bib-0027] Libet, B. (1985). Subjective antedating of a sensory experience and mind‐brain theories: Reply to Honderich (1984). Journal of Theoretical Biology, 114, 563–570. 4021506 10.1016/s0022-5193(85)80043-6

[phy215522-bib-0028] Libet, B. (1999). Do we have free will? Journal of Consciousness Studies, 6, 11.

[phy215522-bib-0029] Libet, B. (2005). Mind time: The temporal factor in consciousness. Harvard University Press.

[phy215522-bib-0030] Libet, B. , Gleason, C. A. , Wright, E. W. , & Pearl, D. K. (1983). Time of conscious intention to act in relation to onset of cerebral activity (readiness‐potential). The unconscious initiation of a freely voluntary act. Brain, 106(Pt 3), 623–642. 6640273 10.1093/brain/106.3.623

[phy215522-bib-0031] Libet, B. , Wright, E. W. , & Gleason, C. A. (1982). Readiness‐potentials preceding unrestricted "spontaneous" vs. pre‐planned voluntary acts. Electroencephalography and Clinical Neurophysiology, 54, 322–335. 6179759 10.1016/0013-4694(82)90181-x

[phy215522-bib-0032] Liotti, M. , Woldorff, M. G. , Perez, R. , & Mayberg, H. S. (2000). An ERP study fo the temporal course of the Stroop color‐word interference effet. Neuropsychologia, 38, 10–711. 10.1016/s0028-3932(99)00106-2 10689046

[phy215522-bib-0033] MacKay, D. M. (1984). Source density analysis of scalp potentials during evaluated action II. Lateral distributions. Experimental Brain Research, 54, 86–94. 6698150 10.1007/BF00235821

[phy215522-bib-0034] Meindl, T. , Schmid, B. C. , Timmann, D. , Kolb, F. P. , & Kutz, D. F. (2012). Contribution of the cerebellum to the coupling of grip force and pull force during an isometric precision grip task. Cerebellum, 11, 167–180. 21717230 10.1007/s12311-011-0293-y

[phy215522-bib-0035] Mele, A. R. (1992). Springs of action: Understanding intentional behavior. Oxford University Press.

[phy215522-bib-0036] Mitzdorf, U. (1985). Current source‐density method and application in cat cerebral cortex: Investigation of evoked potentials and EEG phenomena. Physiological Reviews, 65, 37–100. 3880898 10.1152/physrev.1985.65.1.37

[phy215522-bib-0037] Nicholson, C. , & Freeman, J. A. (1975). Theory of current source‐density analysis and determination of conductivity tensor for anuran cerebellum. Journal of Neurophysiology, 38, 356–368. 805215 10.1152/jn.1975.38.2.356

[phy215522-bib-0038] Nicholson, C. , & Llinas, R. (1975). Real time current source‐density analysis using multi‐electrode array in cat cerebellum. Brain Research, 100, 418–424. 1192185 10.1016/0006-8993(75)90494-1

[phy215522-bib-0039] Nida‐Rümelin, J. (2022). A theory of practical reason. Palgrave Macmillan.

[phy215522-bib-0040] Nunez, P. L. , Silberstein, R. B. , Cadusch, P. J. , Wijesinghe, R. S. , Westdorp, A. F. , & Srinivasan, R. (1994). A theoretical and experimental study of high resolution EEG based on surface Laplacians and cortical imaging. Electroencephalography and Chnical Neurophysiolog, 90, 18. 10.1016/0013-4694(94)90112-0 7509273

[phy215522-bib-0041] Pires, L. , Leitao, J. , Guerrini, C. , & Simones, M. R. (2014). Event‐related brain potentials in the study of inhibition: Cognitive control, source localization and age‐related modulations. Neuropsychology Review, 24, 29. 10.1007/s11065-014-9275-4 25407470

[phy215522-bib-0042] Sattler, J. B. (1999). Das linkshändige kind in der Grundschule (Vol. 8). Auer.

[phy215522-bib-0043] Scherg, M. (1990). Fundamentals of dipole source potential analysis. In F. Grandori & G. Romani (Eds.), Auditory evoked electric and magnetic fields. Topographic mapping and functional localization (Vol. 6, pp. 40–69). Advances in Audiology.

[phy215522-bib-0044] Scherg, M. , & Berg, P. (1991). Use of prior knowledge in brain electromagnetic source analysis. Brain Topography, 4, 8–150. 10.1007/BF01132771 1793688

[phy215522-bib-0045] Scherg, M. , Berg, P. , Nagasato, N. , & Beniczky, S. (2019). Taking the EEG Back into the brain: The power of multiple discrete sources. Frontiers in Neurology, 10, 23. 31481921 10.3389/fneur.2019.00855 PMC6710389

[phy215522-bib-0046] Schlosser, M. E. (2012). Causally efficacious intentions and the sense of agency: In defense of real mental causation. Journal of Theoretical and Philosophical Psychology, 32, 26.

[phy215522-bib-0047] Shibasaki, H. , Barrett, G. , Halliday, E. , & Halliday, A. M. (1980). Components of the movement‐related cortical potential and their scalp topography. Electroencephalography and Clinical Neurophysiology, 49, 213–226. 6158398 10.1016/0013-4694(80)90216-3

[phy215522-bib-0048] Snedecor, G. W. , & Cochran, W. G. (1989). Statistical methods. Iowa State University Press.

[phy215522-bib-0049] Soon, C. S. , Brass, M. , Heinze, H. J. , & Haynes, J. D. (2008). Unconscious determinants of free decisions in the human brain. Nature Neuroscience, 11, 543–545. 18408715 10.1038/nn.2112

[phy215522-bib-0050] Stroop, R. (1935). Studies of interference in serial verbal reactions. Journal of Experimental Psychology, 18(6), 643–662.

[phy215522-bib-0051] Szucs, D. , Soltesz, F. , & White, S. (2009). Motor conflict in Stroop tasks: Direct evidence from single‐trial electro‐myography and electro‐encephalography. Neuroimage, 47, 1960–1973. 19481157 10.1016/j.neuroimage.2009.05.048

[phy215522-bib-0052] Toma, K. , Matsuoka, T. , Immisch, I. , Mima, T. , Waldvogel, D. , Koshy, B. , Hanakawa, T. , Shill, H. , & Hallett, M. (2002). Generators of movement‐related cortical potentials: fMRI‐constrained EEG dipole source analysis. Neuroimage, 17, 161–173. 12482074 10.1006/nimg.2002.1165

[phy215522-bib-0053] Trevena, J. , & Miller, J. (2010). Brain preparation before a voluntary action: Evidence against unconscious movement initiation. Consciousness and Cognition, 19, 447–456. 19736023 10.1016/j.concog.2009.08.006

[phy215522-bib-0054] Vaughan, H. G., Jr. , Costa, L. D. , & Ritter, W. (1968). Topography of the human motor potential. Electroencephalography and Clinical Neurophysiology, 25, 1–10. 4174778 10.1016/0013-4694(68)90080-1

[phy215522-bib-0055] Walter, W. G. , Cooper, R. , Aldridge, V. J. , McCallum, W. C. , & Winter, A. L. (1964). Contingent negative variation: An electric sign of sensorimotor association and expectancy in the human brain. Nature, 203, 380–384. 14197376 10.1038/203380a0

[phy215522-bib-0056] Zhu, J. (2003). Reclaiming volition: An alternative interpretation of Libet's experiment. Journal of Consciousness Studies, 10, 16.

